# Supplementary material for: Trends in Diagnosis and Surgical Treatment of Bone Metastases among Orthopedic Surgeons
Source: J Clin Med. 2022 Jul 23;11(15):4284. doi: 10.3390/jcm11154284 (PMC9341391; doi:10.3390/jcm11154284)
Supplement: Supplementary file 1 [file jcm-11-04284-s001.zip › jcm-1757820-supplementary/File S1 - Survey PL.pdf]

Trendy w postępowaniu diagnostyczno-terapeutycznym u pacjentów z przerzutami do kości długich - badanie ankietowe wśród ortopedów w Polsce

Szanowne Koleżanki, Szanowni Koledzy,

Ankieta ma na celu zbadanie obecnie panujących trendów w diagnostyce, kwalifikowaniu i leczeniu pacjentów ze zmianami przerzutowymi do kości. Podzielona jest ona na 2 części główne - część teoretyczna oraz przypadki kliniczne.

Rozwiązanie całości powinno zająć maksymalnie 15 minut.

Ankieta powstała jako projekt badawczy realizowany w Katedrze i Klinice Ortopedii i Traumatologii Gdańskiego Uniwersytetu Medycznego.

Z góry serdecznie dziękujemy za poświęcony czas i wypełnienie poniższej ankiety.

Jestem świadom, że udział w anonimowej ankiecie jest dobrowolny i zgadzam się na udział w badaniu przechodząc do dalszej części ankiety.

---

*Wymagane

Podstawowe informacje - profil

1. Płeć

Zaznacz tylko jedną odpowiedź.

- ◯ Mężczyzna
- ◯ Kobieta

2. Jakie masz doświadczenie w pracy jako Ortopeda (wliczając okres szkolenia specjalizacyjnego)? *

Zaznacz tylko jedną odpowiedź.

- 0-5 lat
- 6-10 lat
- 11-20 lat
- >20 lat

3. W jakiej instytucji w ramach pracy jako lekarz ortopeda wykonujesz obecnie (większość) swoją pracę? *

Zaznacz tylko jedną odpowiedź.

- Szpital Uniwersytecki/Szpital o najwyższym stopniu referencyjności
- Szpital powiatowy
- Prywatna praktyka
- Inne: ____________________

4. Jak często wykonujesz operacje z powodu zmian przerzutowych w kościach (średnia liczba przez rok)? *

Zaznacz tylko jedną odpowiedź.

- 0-5
- 6-10
- 11-20
- >20

5. Główny obszar pracy i zainteresowań: (możliwa więcej niż jedna odpowiedź; max 3 odpowiedzi) *

Zaznacz wszystkie właściwe odpowiedzi.

- [ ] Ortopedia ogólna
- [ ] Traumatologia
- [ ] Onkologia Narządu Ruchu
- [ ] Artroplastyka stawów
- [ ] Artroskopia i ortopedia małoinwazyjna
- [ ] Ortopedia kręgosłupa
- [ ] Ortopedia pediatryczna
- [ ] Chirurgia ręki
- [ ] Chirurgia w obrębie stopy

Inne: [ ] ______________________

6. Jak pewnie czujesz się wykonując zabiegi operacyjne przerzutowych guzów kości? *

Zaznacz tylko jedną odpowiedź.

	1	2	3	4	5	
Zdecydowanie niepewnie	( )	( )	( )	( )	( )	Zdecydowanie pewnie

Diagnostyka i kwalifikowanie do zabiegu

7. Jakie preferujesz postępowanie z pacjentem zgłaszającym się na izbę przyjęć/do poradni ortopedycznej, z podejrzeniem przerzutu do kości? *

Zaznacz tylko jedną odpowiedź.

- ( ) Skierowanie do poradni onkologicznej
- ( ) Skierowanie do ośrodka ortopedycznego o wyższej referencyjności
- ( ) Przyjęcie na oddział i wykonanie kwalifikacji do leczenia\biopsji
- ( ) Inne: ______________________

8. Jakich skal/klasyfikacji używasz w przypadku kwalifikowania pacjenta do zabiegu (możesz wybrać więcej niż jedną odpowiedź)? *

Zaznacz wszystkie właściwe odpowiedzi.

- [ ] Bollen Prognostic Scale
- [ ] The Modified Bauer Score
- [ ] Forsberga (Bayesian-Estimated Tools for Survival - BETS)
- [ ] Katagiri Score
- [ ] Mirels Classification
- [ ] Skala Karnofsky'ego
- [ ] Musculoskeletal Tumor Society Scoring system (MSTS)
- [ ] Capanna classification
- [ ] Nie korzystam ze skal

Inne: [ ] ____________________

9. Czy korzystasz z aplikacji PATHFx do oceny potencjalnej długości życia pacjentów ze zmianami przerzutowymi w kościach (www.pathfx.org)? *

Zaznacz tylko jedną odpowiedź.

- ( ) Tak
- ( ) Nie - znam tą aplikację, ale z niej nie korzystam
- ( ) Nie - nie znałem wcześniej tej aplikacji

10. Jaki rodzaj biopsji preferujesz przy podejrzeniu przerzutu do kości? *

Zaznacz tylko jedną odpowiedź.

- ( ) Biopsja gruboigłowa
- ( ) Trepanobiopsja
- ( ) Biopsja operacyjna (otwarta)
- ( ) Nigdy nie wykonuję biopsji przy podejrzeniu przerzutu do kości
- ( ) Inne: ____________________

11. Jak bardzo zgadzasz się z poniższym stwierdzeniem - Pacjenci z pojedynczą zmianą, podejrzaną o przerzut do kości, powinni mieć wykonaną biopsję w celu wykluczenia pierwotnych nowotworów kości? *

Zaznacz tylko jedną odpowiedź.

	1	2	3	4	5	
Zdecydowanie się nie zgadzam	☐	☐	☐	☐	☐	Zdecydowanie się zgadzam

12. Z jakich metod obrazowania korzystasz w swojej praktyce przy kwalifikowaniu pacjenta do leczenia operacyjnego guza przerzutowego kości? (wielokrotnego wyboru) *

Zaznacz wszystkie właściwe odpowiedzi.

- [ ] RTG
- [ ] Tomografia komputerowa (CT)
- [ ] Rezonans Magnetyczny (MRI)
- [ ] Scyntygrafia kości
- [ ] PET-CT

Inne: [ ] ____________

Jak ważne są dla Ciebie poniższe objawy/czynniki, przy kwalifikowaniu pacjenta do leczenia operacyjnego z powodu przerzutów do kości?

13. Stopień nasilenia bólu *

Zaznacz tylko jedną odpowiedź.

	1	2	3	4	5	
Zdecydowanie nieważne	☐	☐	☐	☐	☐	Zdecydowanie ważne

14. Liczba przerzutów do kości (pojedynczy/mnogie) *

Zaznacz tylko jedną odpowiedź.

	1	2	3	4	5	
Zdecydowanie nieważne	⬭	⬭	⬭	⬭	⬭	Zdecydowanie ważne

15. Wielkość zmiany oraz stopień destrukcji kości *

Zaznacz tylko jedną odpowiedź.

	1	2	3	4	5	
Zdecydowanie nieważne	⬭	⬭	⬭	⬭	⬭	Zdecydowanie ważne

16. Wystąpienie złamania patologicznego *

Zaznacz tylko jedną odpowiedź.

	1	2	3	4	5	
Zdecydowanie nieważne	⬭	⬭	⬭	⬭	⬭	Zdecydowanie ważne

17. Duże ryzyko wystąpienia złamania (zajęcie ponad 50% obwodu warstwy korowej) *

Zaznacz tylko jedną odpowiedź.

	1	2	3	4	5	
Zdecydowanie nieważne	⬭	⬭	⬭	⬭	⬭	Zdecydowanie ważne

18. Przewidywana długość przeżycia pacjenta *

Zaznacz tylko jedną odpowiedź.

	1	2	3	4	5	
Zdecydowanie nieważne	◯	◯	◯	◯	◯	Zdecydowanie ważne

19. Rodzaj nowotworu pierwotnego *

Zaznacz tylko jedną odpowiedź.

	1	2	3	4	5	
Zdecydowanie nieważne	◯	◯	◯	◯	◯	Zdecydowanie ważne

20. Obecność zmian przerzutowych w narządach trzewnych (visceral metastases) *

Zaznacz tylko jedną odpowiedź.

	1	2	3	4	5	
Zdecydowanie nieważne	◯	◯	◯	◯	◯	Zdecydowanie ważne

21. Ocena funkcjonalna i mobilność pacjenta *

Zaznacz tylko jedną odpowiedź.

	1	2	3	4	5	
Zdecydowanie nieważne	◯	◯	◯	◯	◯	Zdecydowanie ważne

22. Ocena jakości życia pacjenta *

Zaznacz tylko jedną odpowiedź.

	1	2	3	4	5	
Zdecydowanie nieważne	⬭	⬭	⬭	⬭	⬭	Zdecydowanie ważne

23. Czas od postawienia rozpoznania nowotworu pierwotnego do wykrycia zmiany przerzutowej *

Zaznacz tylko jedną odpowiedź.

	1	2	3	4	5	
Zdecydowanie nieważne	⬭	⬭	⬭	⬭	⬭	Zdecydowanie ważne

24. Stężenie hemoglobiny w osoczu *

Zaznacz tylko jedną odpowiedź.

	1	2	3	4	5	
Zdecydowanie nieważne	⬭	⬭	⬭	⬭	⬭	Zdecydowanie ważne

Leczenie

25. Czy stosujesz/zalecasz przed operacją przerzutów do kości raka nerki lub tarczycy zabieg embolizacji naczyń krwionośnych guza przerzutowego, przy bogatym jego unaczynieniu? *

Zaznacz tylko jedną odpowiedź.

	1	2	3	4	5	
Zdecydowanie nie	⬭	⬭	⬭	⬭	⬭	Zdecydowanie tak

26. Gdzie kierujesz dalej pacjenta po leczeniu operacyjnym guzów przerzutowych do kości? *

Zaznacz tylko jedną odpowiedź.

- Poradnia onkologiczna
- Lekarz POZ
- Poradnia ortopedyczna
- Inne: ____________

27. Jakiej metody wypełnienia ubytków kości używasz przy leczeniu operacyjnym przerzutów do kości? *

Zaznacz wszystkie właściwe odpowiedzi.

- [ ] Cement kostny
- [ ] Mrożony przeszczep allogeniczny
- [ ] Przeszczep autogeniczny z talerza kości biodrowej
- [ ] Unaczyniony przeszczep strzałkowy
- Inne: [ ] ____________

Przypadki kliniczne

W tej części prosimy o zapoznanie się z przykładowymi przypadkami klinicznymi i wybranie metody leczenia, którą zastosowaliby Państwo w swojej praktyce.

Prezentowane zdjęcia mają charakter poglądowy. Prosimy o kierowanie się głównie informacjami podanymi w tekście przy wyborze leczenia. Przypadki mogą różnić się jedynie pojedynczymi dystraktorami (głównie 'przewidywana długość przeżycia'), dlatego prosimy o uważne zapoznanie się z opisem.

Przypadek 1

Pacjentka 60 r.ż.
- złamanie patologiczne
- rak nerki z obecnością przerzutu do trzonu kości udowej (potwierdzony w badaniu histopatologicznym)
- silne dolegliwości bólowe, powodujące niepełnosprawność
- przewidywana długość życia powyżej 12 miesięcy

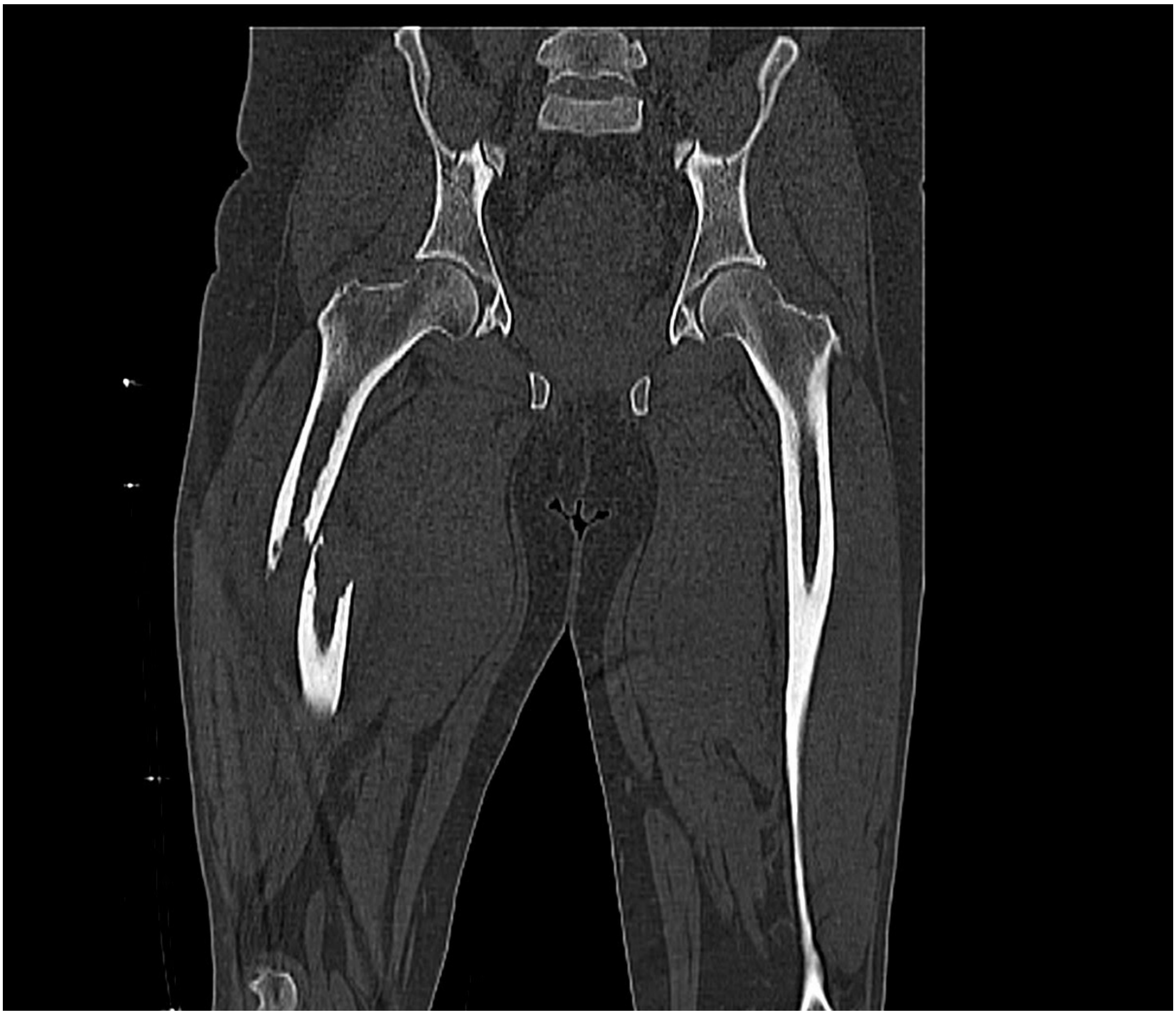

28. Jakie leczenie zastosujesz? *

Zaznacz tylko jedną odpowiedź.

- Gwóźdź śródszpikowy z resekcją guza z zastosowaniem cementu kostnego
- Gwóźdź śródszpikowy bez resekcji guza
- Płyta i śruby
- Endoproteza modularna trzonu kości z wycięciem zmiany
- Brak wskazań do leczenia operacyjnego
- Inne: ____________________

Przypadek 2

Pacjentka 60 r.ż.
- złamanie patologiczne
- rak nerki z obecnością przerzutu do trzonu kości udowej (potwierdzony w badaniu histopatologicznym)
- silne dolegliwości bólowe, powodujące niepełnosprawność
- przewidywana długość życia poniżej 6 miesięcy

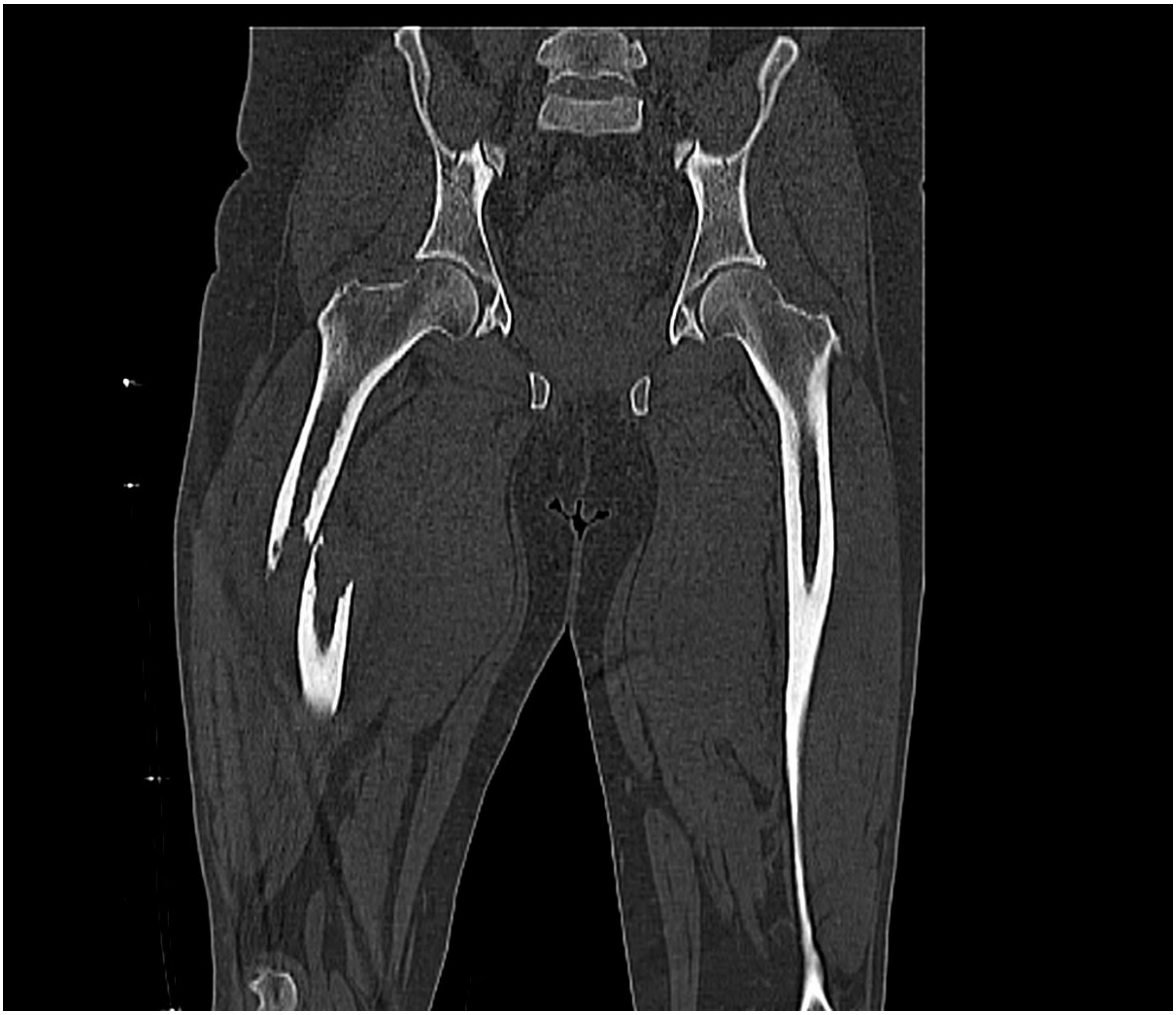

29. Jakie leczenie zastosujesz? *

Zaznacz tylko jedną odpowiedź.

- Gwóźdź śródszpikowy z resekcją guza z zastosowaniem cementu kostnego
- Gwóźdź śródszpikowy bez resekcji guza
- Płyta i śruby
- Endoproteza modularna trzonu kości z wycięciem zmiany
- Brak wskazań do leczenia operacyjnego
- Inne: ____________________

Przypadek 3

Pacjentka 60 r.ż.
- bez cech złamania patologicznego na RTG, wysokie ryzyko złamania patologicznego
- rak nerki ze zmianą przerzutową do kości ramiennej (potwierdzony w badaniu histopatologicznym)
- silne dolegliwości bólowe, powodujące niepełnosprawność
- przewidywana długość życia powyżej 12 miesięcy

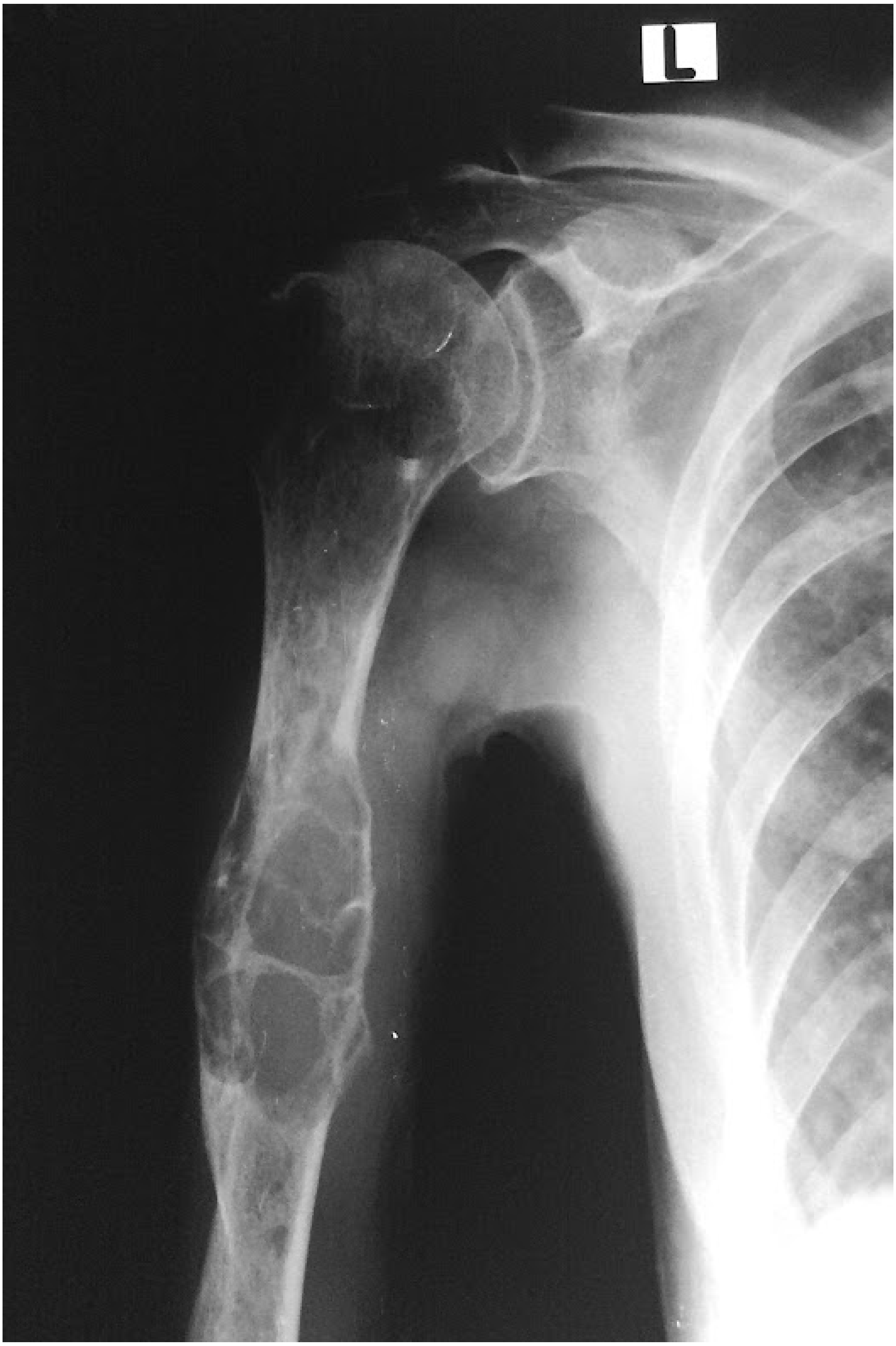
L

30. Jakie leczenie zastosujesz? *

Zaznacz tylko jedną odpowiedź.

- Gwóźdź śródszpikowy z resekcją guza z zastosowaniem cementu kostnego
- Gwóźdź śródszpikowy bez resekcji guza
- Płyta i śruby
- Endoproteza modularna trzonu kości z wycięciem zmiany
- Brak wskazań do leczenia operacyjnego
- Inne: __________

Przypadek 4

Pacjent 60 r.ż.
- bez cech złamania patologicznego na RTG, wysokie ryzyko złamania patologicznego
- rak nerki ze zmianą przerzutową do kości ramiennej (potwierdzony w badaniu histopatologicznym)
- silne dolegliwości bólowe, powodujące niepełnosprawność
- przewidywana długość życia poniżej 6 miesięcy

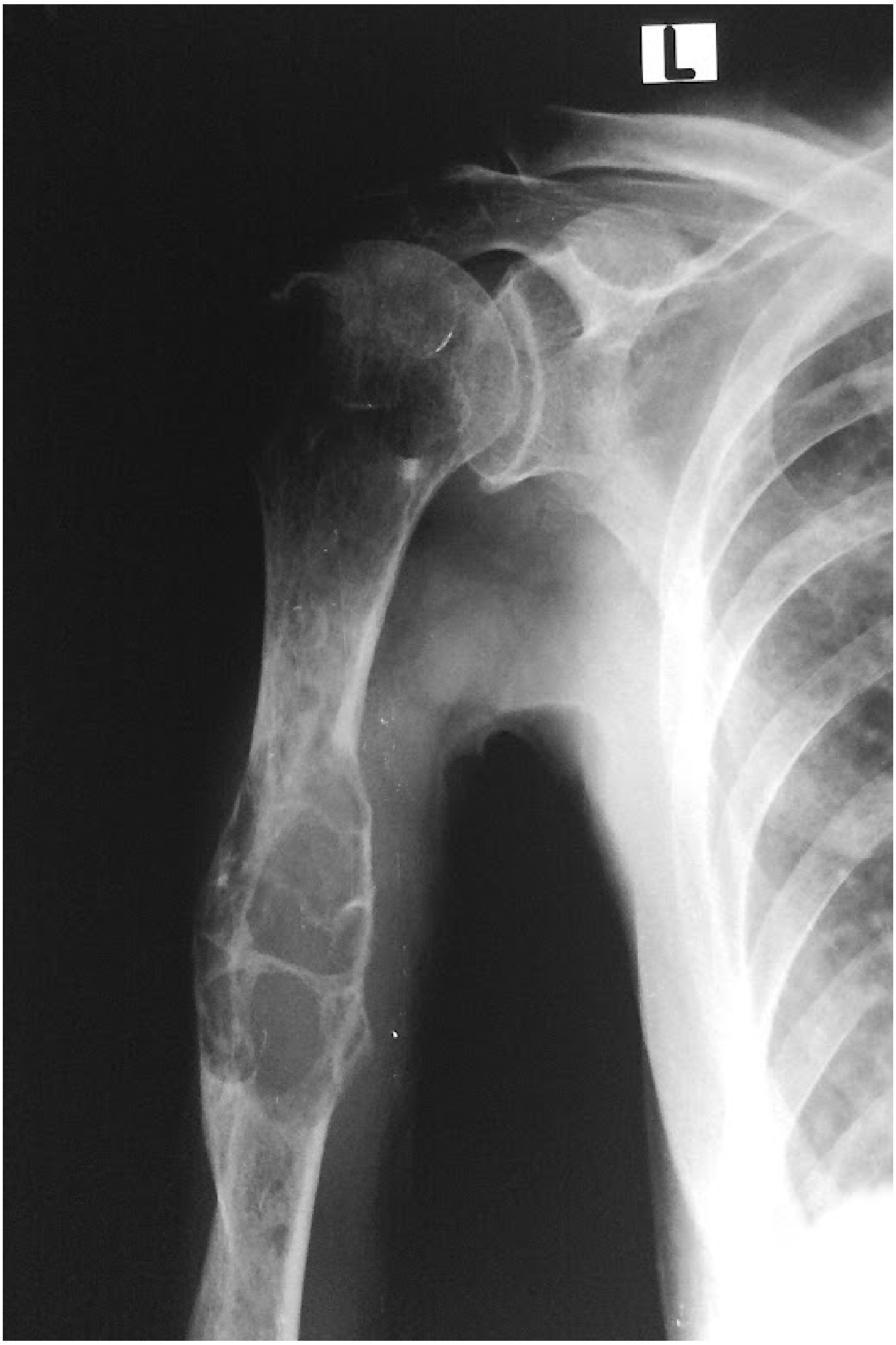
L

31. Jakie leczenie zastosujesz? *

Zaznacz tylko jedną odpowiedź.

- Gwóźdź śródszpikowy z resekcją guza z zastosowaniem cementu kostnego
- Gwóźdź śródszpikowy bez resekcji guza
- Płyta i śruby
- Endoproteza modularna trzonu kości z wycięciem zmiany
- Brak wskazań do leczenia operacyjnego
- Inne: ______________________

Przypadek 5

Pacjentka 60 r.ż.
- złamanie patologiczne
- rak piersi z obecnością przerzutu do trzonu kości udowej (potwierdzony w badaniu histopatologicznym)
- silne dolegliwości bólowe, powodujące niepełnosprawność
- przewidywana długość życia powyżej 12 miesięcy

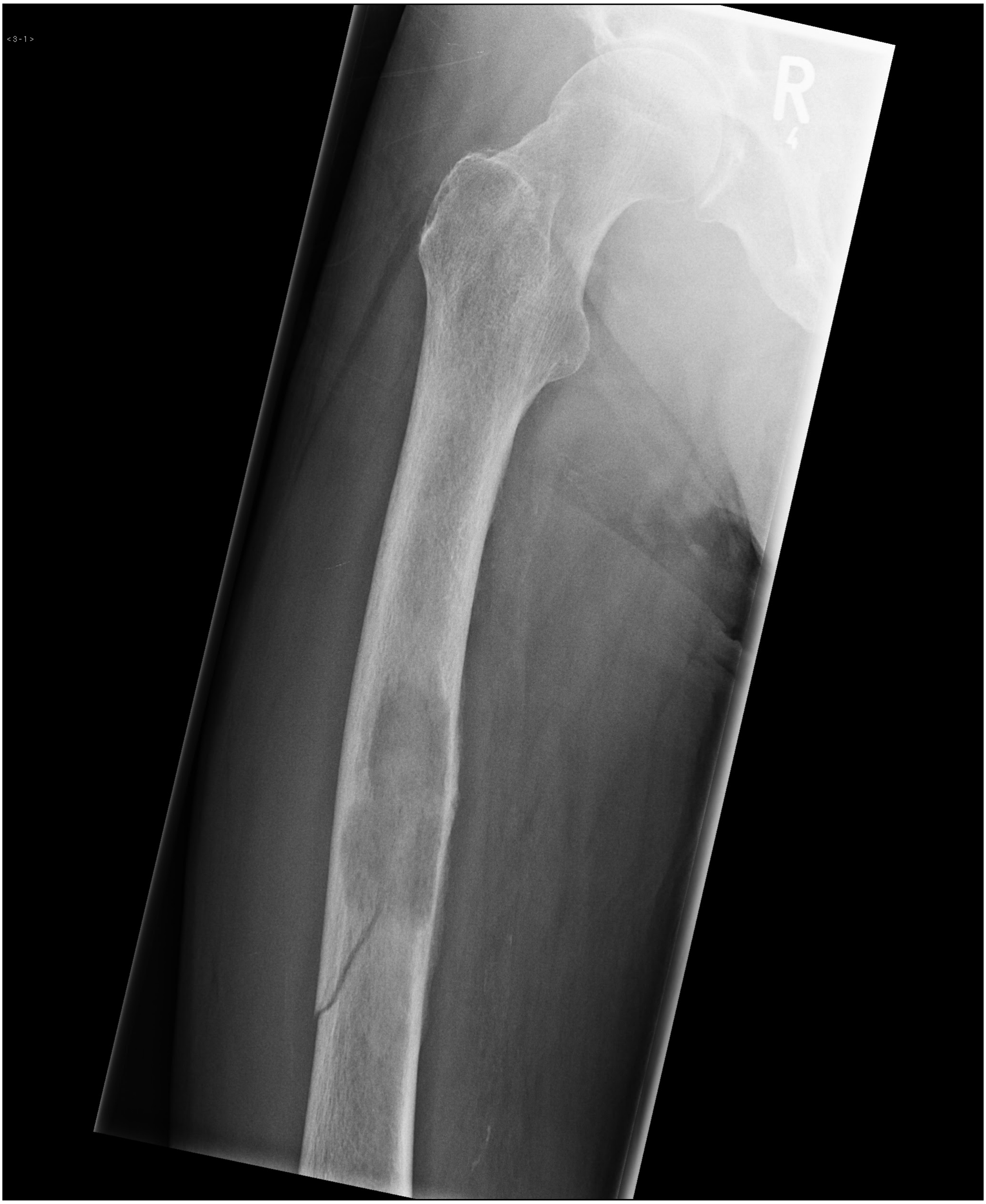
R

32. Jakie leczenie zastosujesz? *

Zaznacz tylko jedną odpowiedź.

- Gwóźdź śródszpikowy z resekcją guza z zastosowaniem cementu kostnego
- Gwóźdź śródszpikowy bez resekcji guza
- Płyta i śruby
- Endoproteza modularna trzonu kości z wycięciem zmiany
- Brak wskazań do leczenia operacyjnego
- Inne: ____________________

Przypadek 6

Pacjentka 60 r.ż.
- złamanie patologiczne
- rak piersi z obecnością przerzutu do trzonu kości udowej (potwierdzony w badaniu histopatologicznym)
- silne dolegliwości bólowe, powodujące niepełnosprawność
- przewidywana długość życia poniżej 6 miesięcy

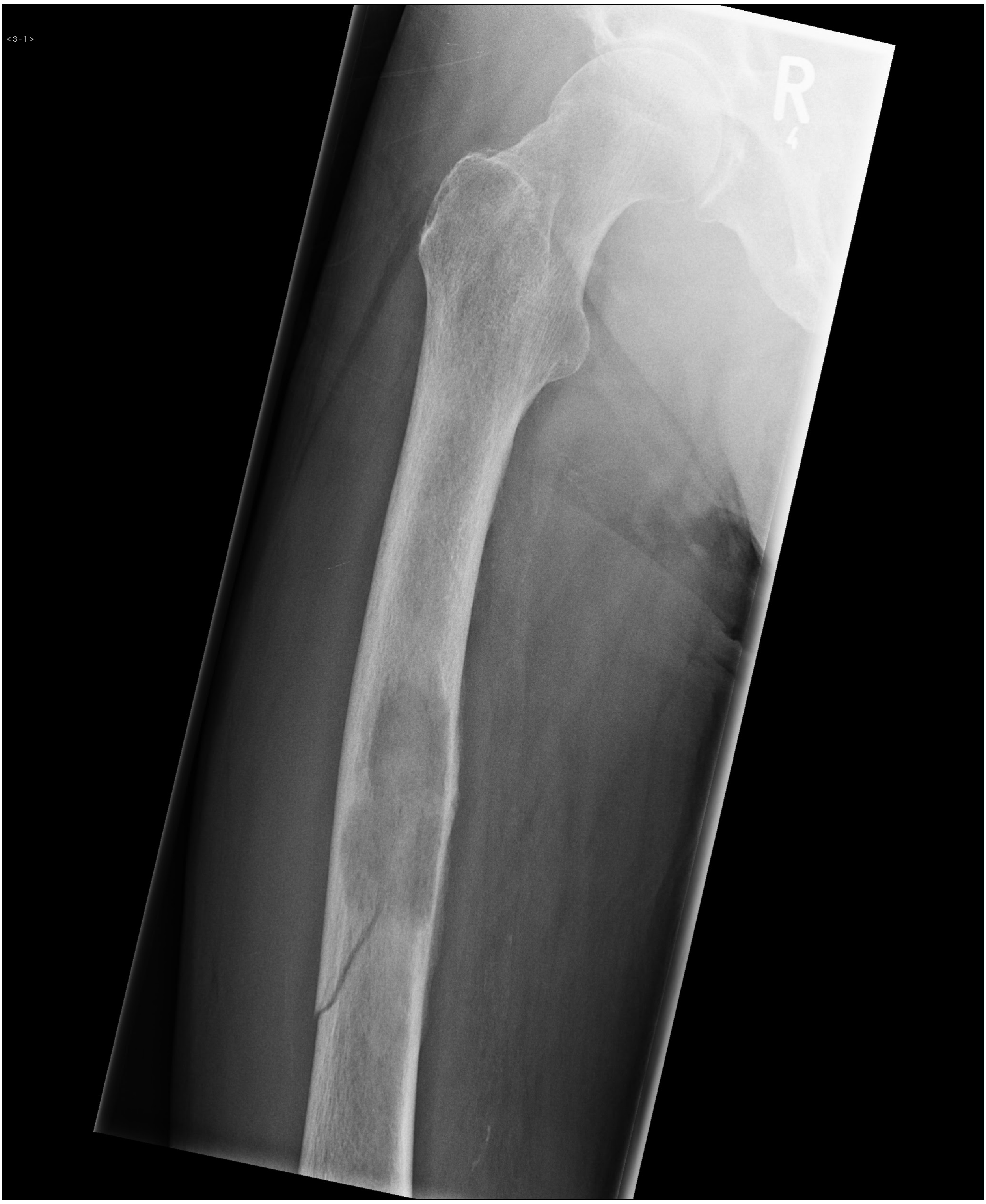
R

33. Jakie leczenie zastosujesz? *

Zaznacz tylko jedną odpowiedź.

- Gwóźdź śródszpikowy z resekcją guza z zastosowaniem cementu kostnego
- Gwóźdź śródszpikowy bez resekcji guza
- Płyta i śruby
- Endoproteza modularna trzonu kości z wycięciem zmiany
- Brak wskazań do leczenia operacyjnego
- Inne: ____________________

Przypadek 7

Pacjentka 60 r.ż.
- złamanie patologiczne
- rak nerki z obecnością przerzutu do trzonu kości ramiennej (potwierdzony w badaniu histopatologicznym)
- silne dolegliwości bólowe, powodujące niepełnosprawność
- przewidywana długość życia powyżej 12 miesięcy

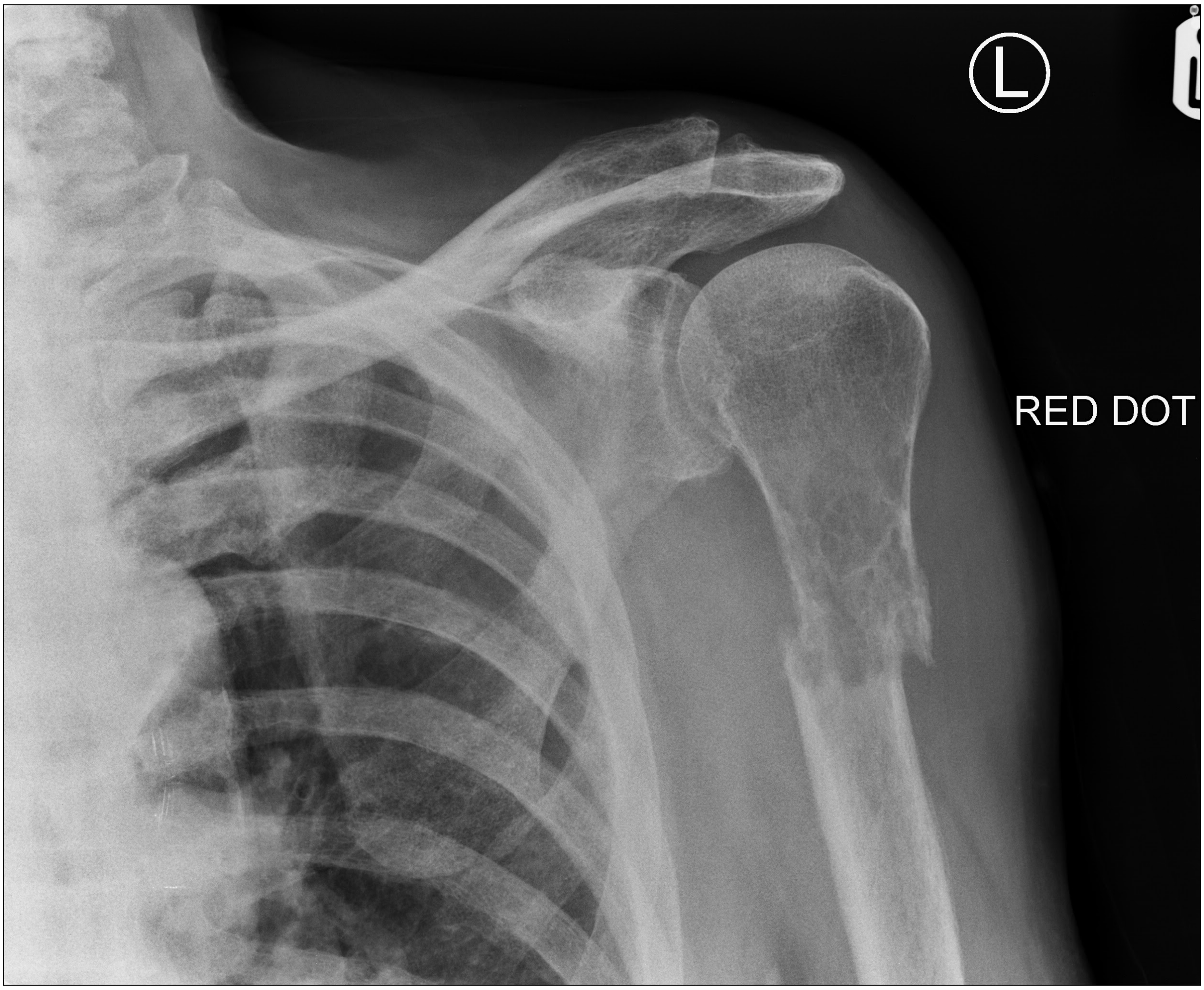

34. Jakie leczenie zastosujesz? *

Zaznacz tylko jedną odpowiedź.

- Gwóźdź śródszpikowy z resekcją guza z zastosowaniem cementu kostnego
- Gwóźdź śródszpikowy bez resekcji guza
- Płyta i śruby
- Endoproteza modularna trzonu kości z wycięciem zmiany
- Brak wskazań do leczenia operacyjnego
- Inne: ______________________

Przypadek 8

Pacjentka 60 r.ż.
- złamanie patologiczne
- rak nerki z obecnością przerzutu do trzonu kości ramiennej (potwierdzony w badaniu histopatologicznym)
- silne dolegliwości bólowe, powodujące niepełnosprawność
- przewidywana długość życia poniżej 6 miesięcy

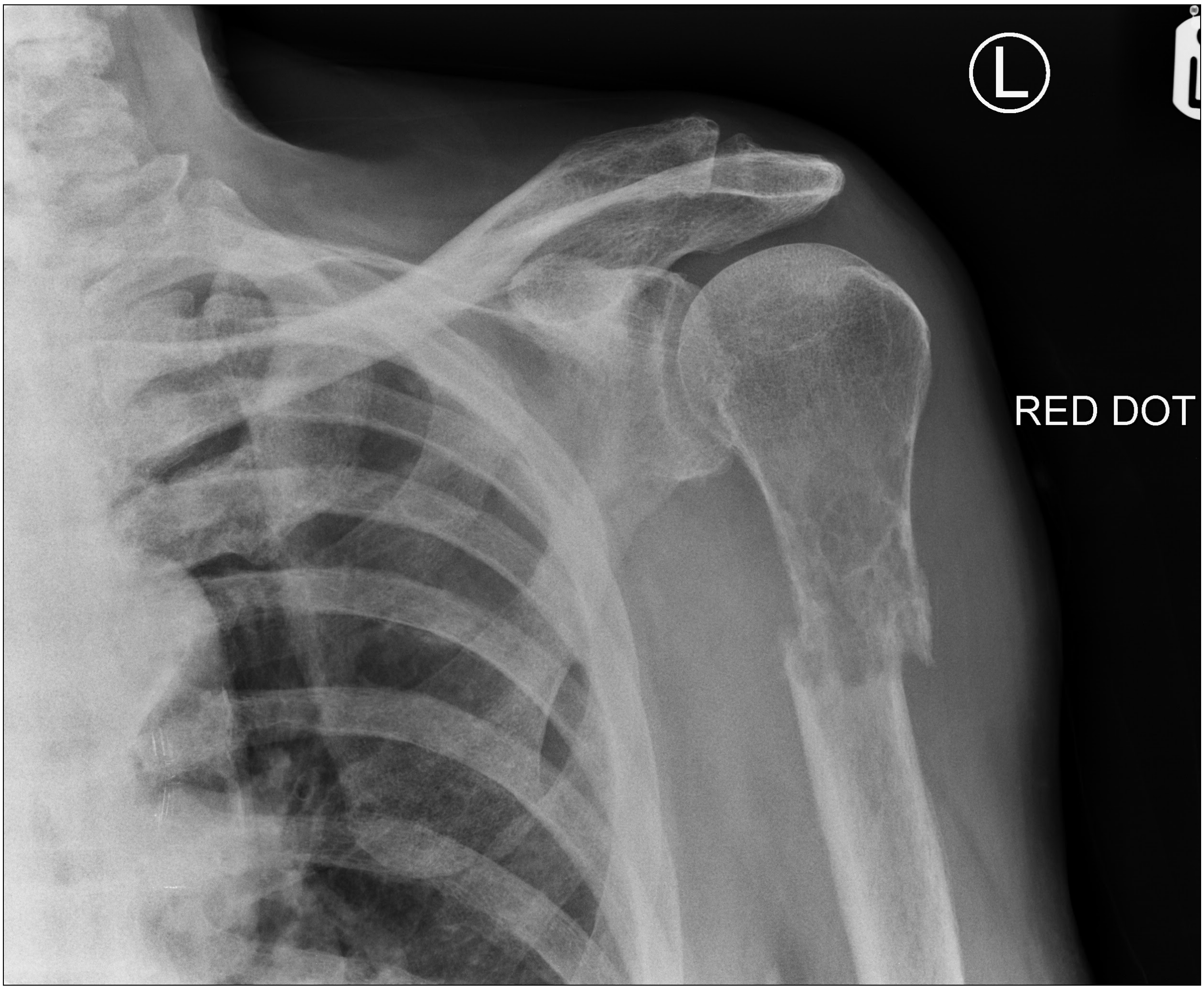

35. Jakie leczenie zastosujesz? *

Zaznacz tylko jedną odpowiedź.

- Gwóźdź śródszpikowy z resekcją guza z zastosowaniem cementu kostnego
- Gwóźdź śródszpikowy bez resekcji guza
- Płyta i śruby
- Endoproteza modularna trzonu kości z wycięciem zmiany
- Brak wskazań do leczenia operacyjnego
- Inne: ______________________

Przypadek 9

Pacjent 60 r.ż.
- bez cech złamania patologicznego na RTG, wysokie ryzyko złamania patologicznego
- rak nerki ze zmianą przerzutową do kości udowej (potwierdzony w badaniu histopatologicznym)
- silne dolegliwości bólowe, powodujące niepełnosprawność
- przewidywana długość życia powyżej 12 miesięcy

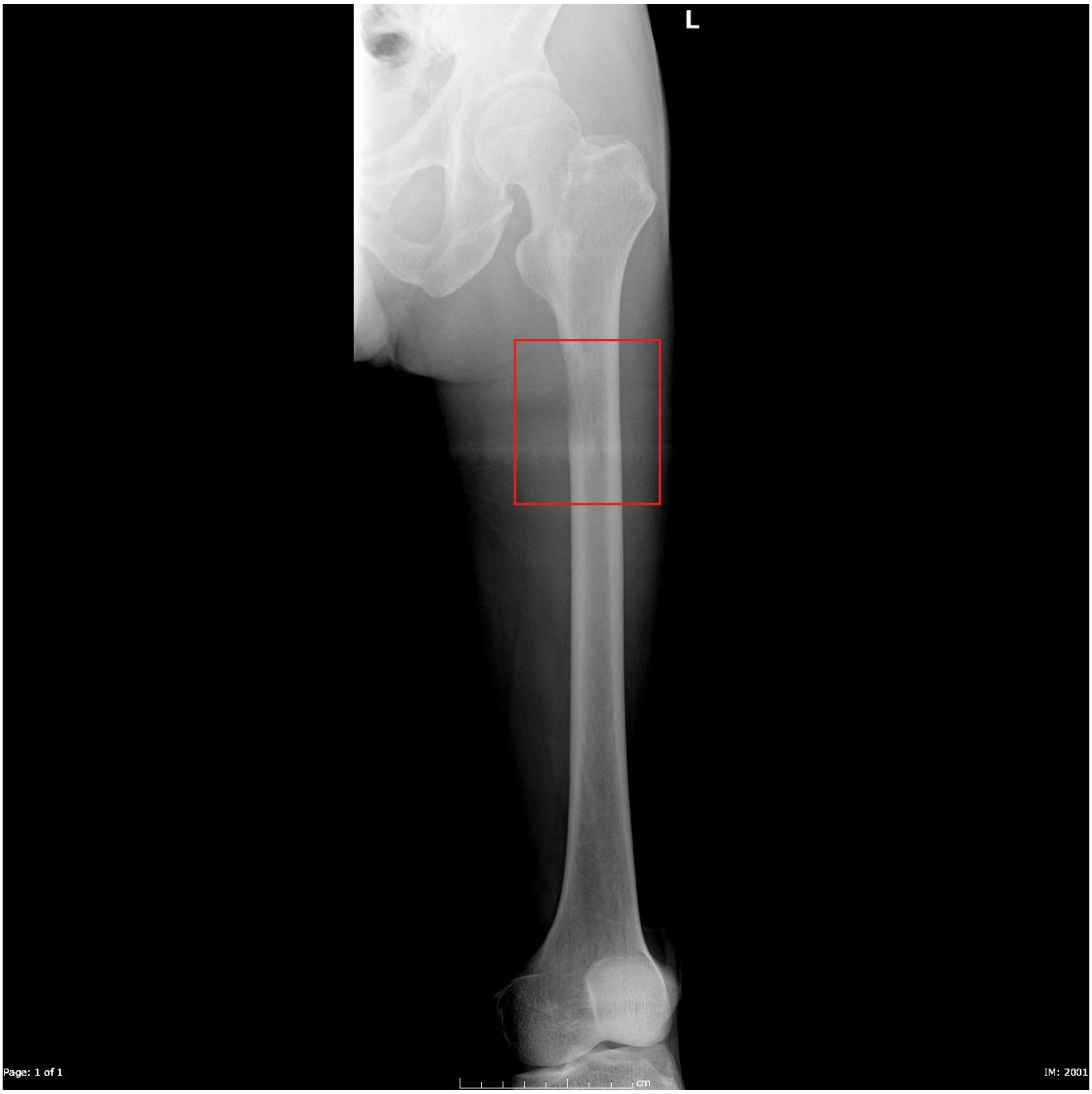

36. Jakie leczenie zastosujesz? *

Zaznacz tylko jedną odpowiedź.

- Gwóźdź śródszpikowy z resekcją guza z zastosowaniem cementu kostnego
- Gwóźdź śródszpikowy bez resekcji guza
- Płyta i śruby
- Endoproteza modularna trzonu kości z wycięciem zmiany
- Brak wskazań do leczenia operacyjnego
- Inne: ______________________

Przypadek 10

Pacjent 60 r.ż.
- bez cech złamania patologicznego na RTG, wysokie ryzyko złamania patologicznego
- rak nerki ze zmianą przerzutową do kości udowej (potwierdzony w badaniu histopatologicznym)
- silne dolegliwości bólowe, powodujące niepełnosprawność
- przewidywana długość życia poniżej 6 miesięcy

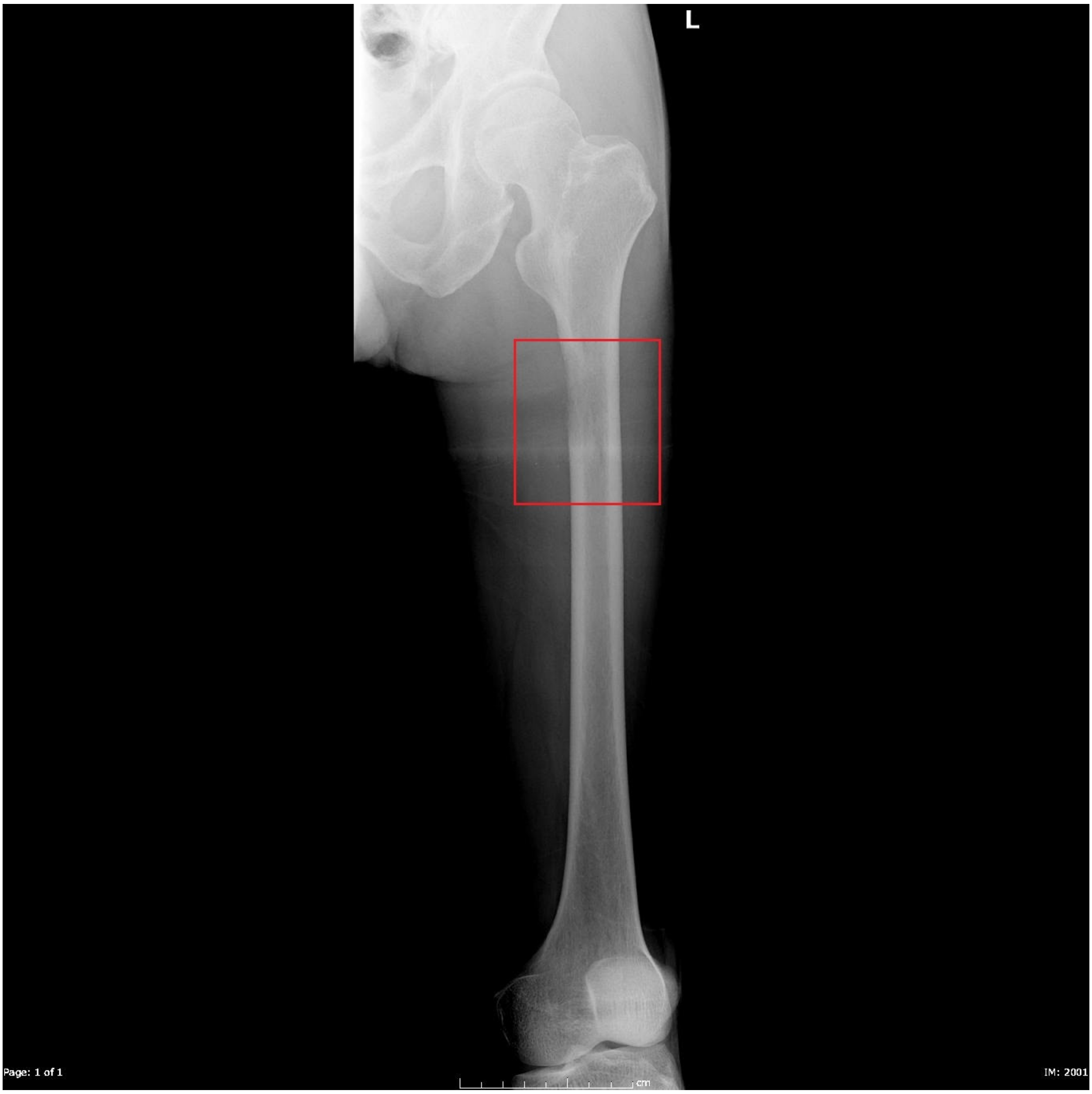

37. Jakie leczenie zastosujesz? *

Zaznacz tylko jedną odpowiedź.

- Gwóźdź śródszpikowy z resekcją guza z zastosowaniem cementu kostnego
- Gwóźdź śródszpikowy bez resekcji guza
- Płyta i śruby
- Endoproteza modularna trzonu kości z wycięciem zmiany
- Brak wskazań do leczenia operacyjnego
- Inne: ______________________

Przypadek 11

Pacjentka 60 r.ż.
- bez cech złamania patologicznego na RTG, wysokie ryzyko złamania patologicznego
- rak piersi ze zmianą przerzutową do trzonu kości ramiennej (potwierdzony w badaniu histopatologicznym)
- silne dolegliwości bólowe, powodujące niepełnosprawność
- przewidywana długość życia powyżej 12 miesięcy

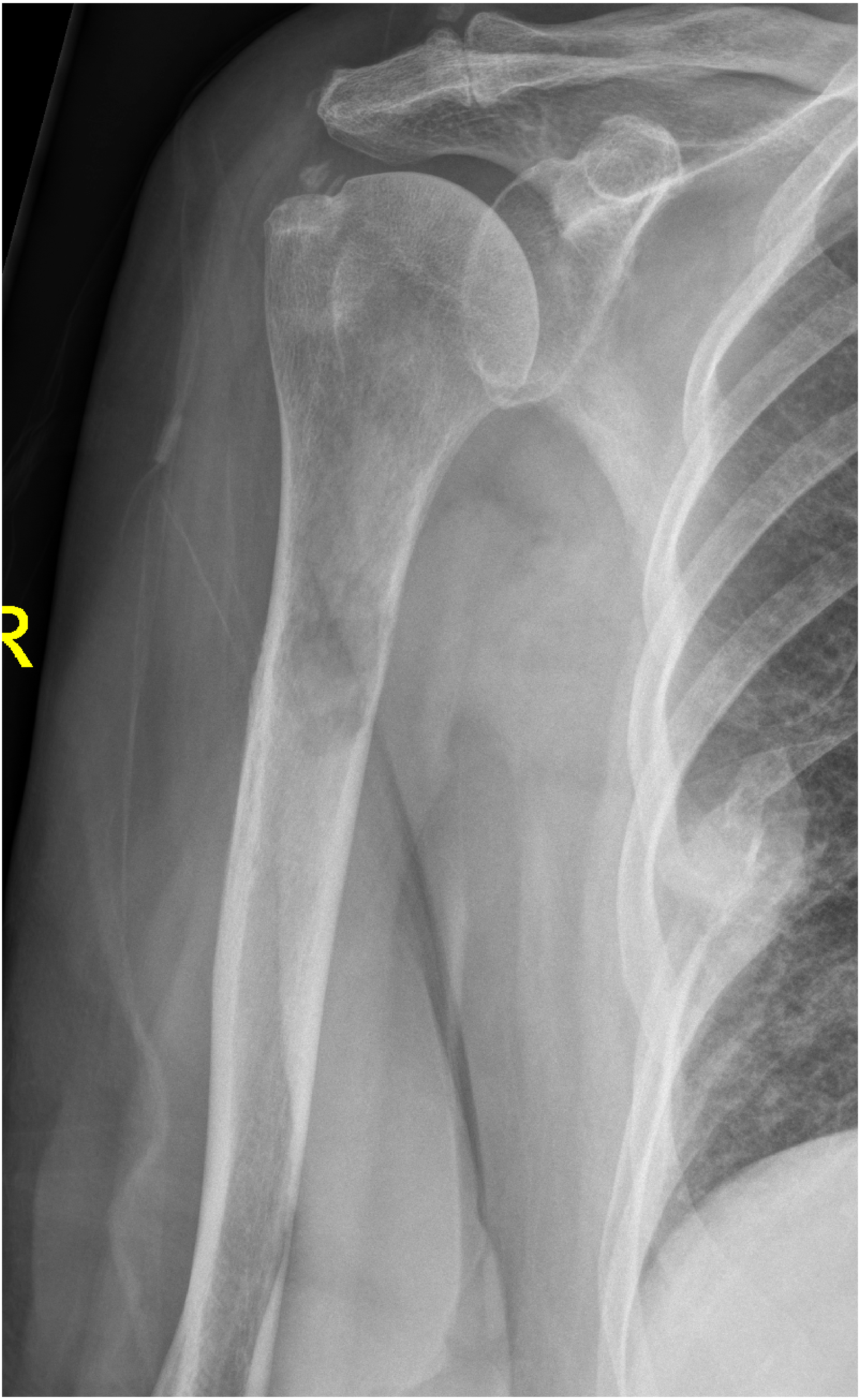
R

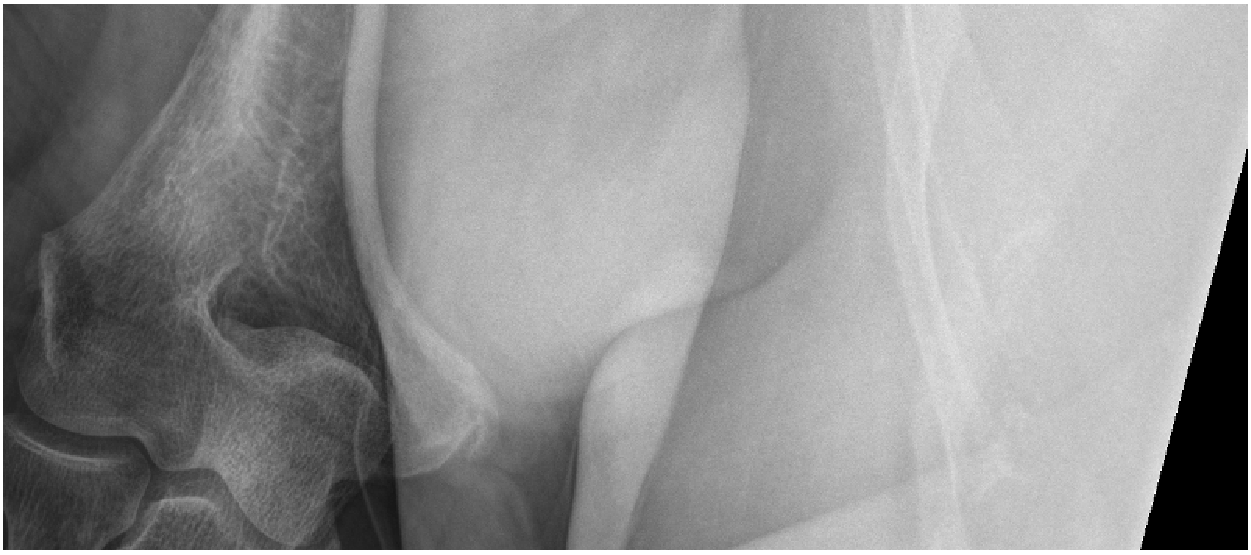

38. Jakie leczenie zastosujesz? *

Zaznacz tylko jedną odpowiedź.

- Gwóźdź śródszpikowy z resekcją guza z zastosowaniem cementu kostnego
- Gwóźdź śródszpikowy bez resekcji guza
- Płyta i śruby
- Endoproteza modularna trzonu kości z wycięciem zmiany
- Brak wskazań do leczenia operacyjnego
- Inne: ____________________

Przypadek 12

Pacjentka 60 r.ż.
- bez cech złamania patologicznego na RTG, wysokie ryzyko złamania patologicznego
- rak piersi ze zmianą przerzutową do trzonu kości ramiennej (potwierdzony w badaniu histopatologicznym)
- silne dolegliwości bólowe, powodujące niepełnosprawność
- przewidywana długość życia poniżej 6 miesięcy

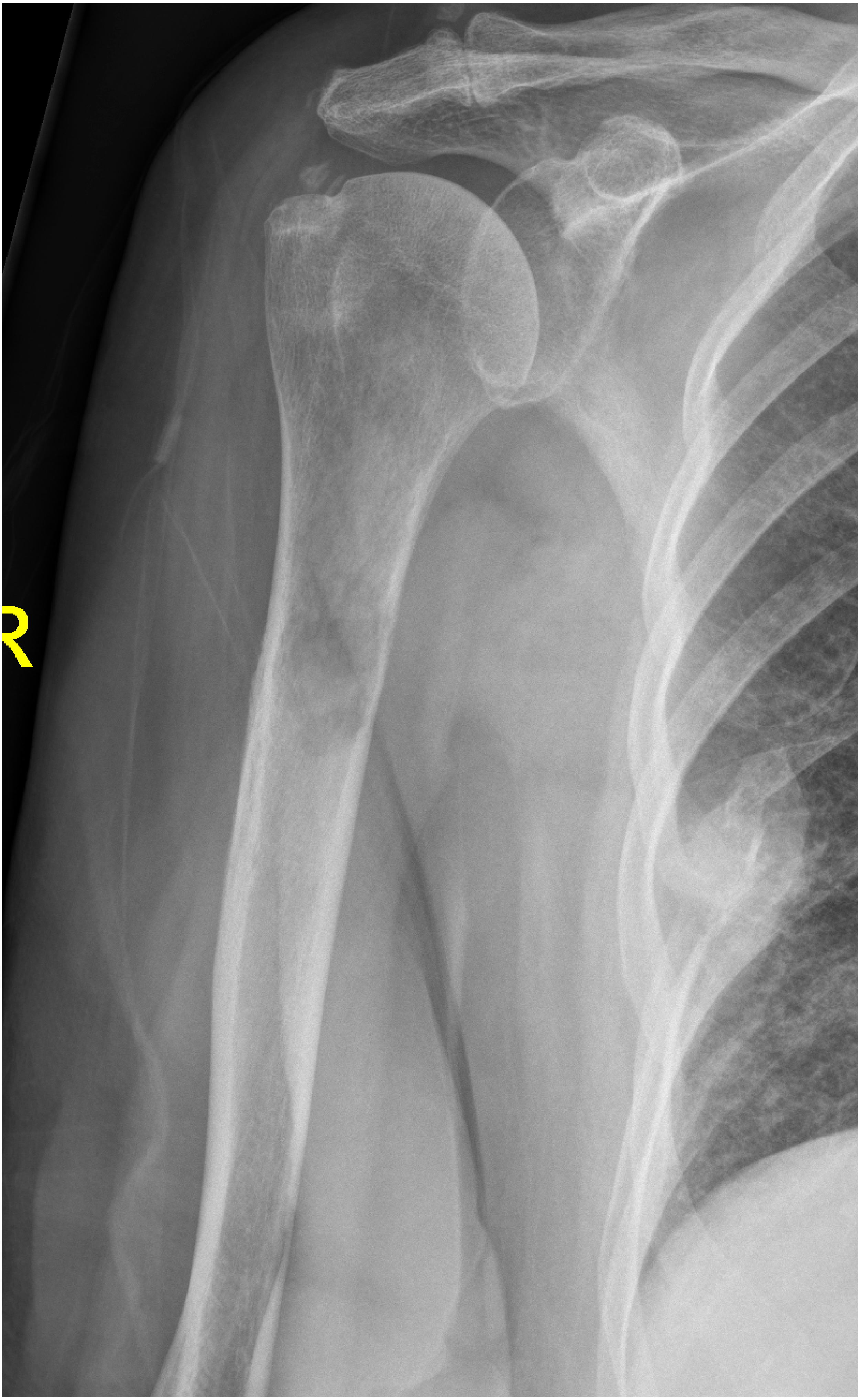
R

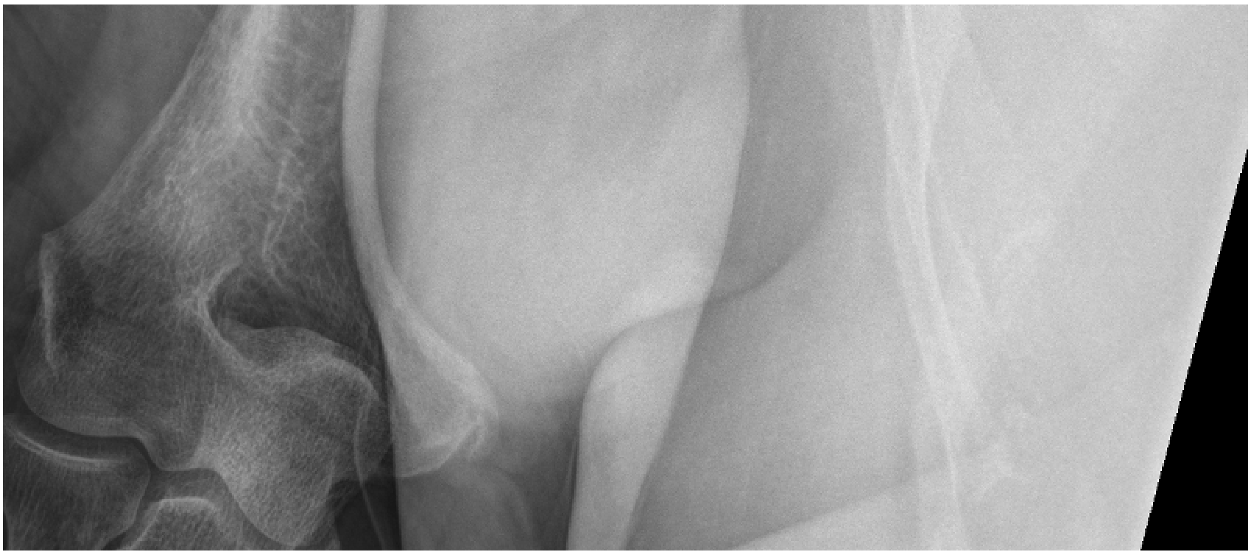

39. Jakie leczenie zastosujesz? *

Zaznacz tylko jedną odpowiedź.

- Gwóźdź śródszpikowy z resekcją guza z zastosowaniem cementu kostnego
- Gwóźdź śródszpikowy bez resekcji guza
- Płyta i śruby
- Endoproteza modularna trzonu kości z wycięciem zmiany
- Brak wskazań do leczenia operacyjnego
- Inne: ______________________

Przypadek 13

Pacjentka 60 r.ż.
- złamanie patologiczne
- rak piersi ze zmianą przerzutową do trzonu kości ramiennej (potwierdzony w badaniu histopatologicznym)
- silne dolegliwości bólowe, powodujące niepełnosprawność
- przewidywana długość życia powyżej 12 miesięcy

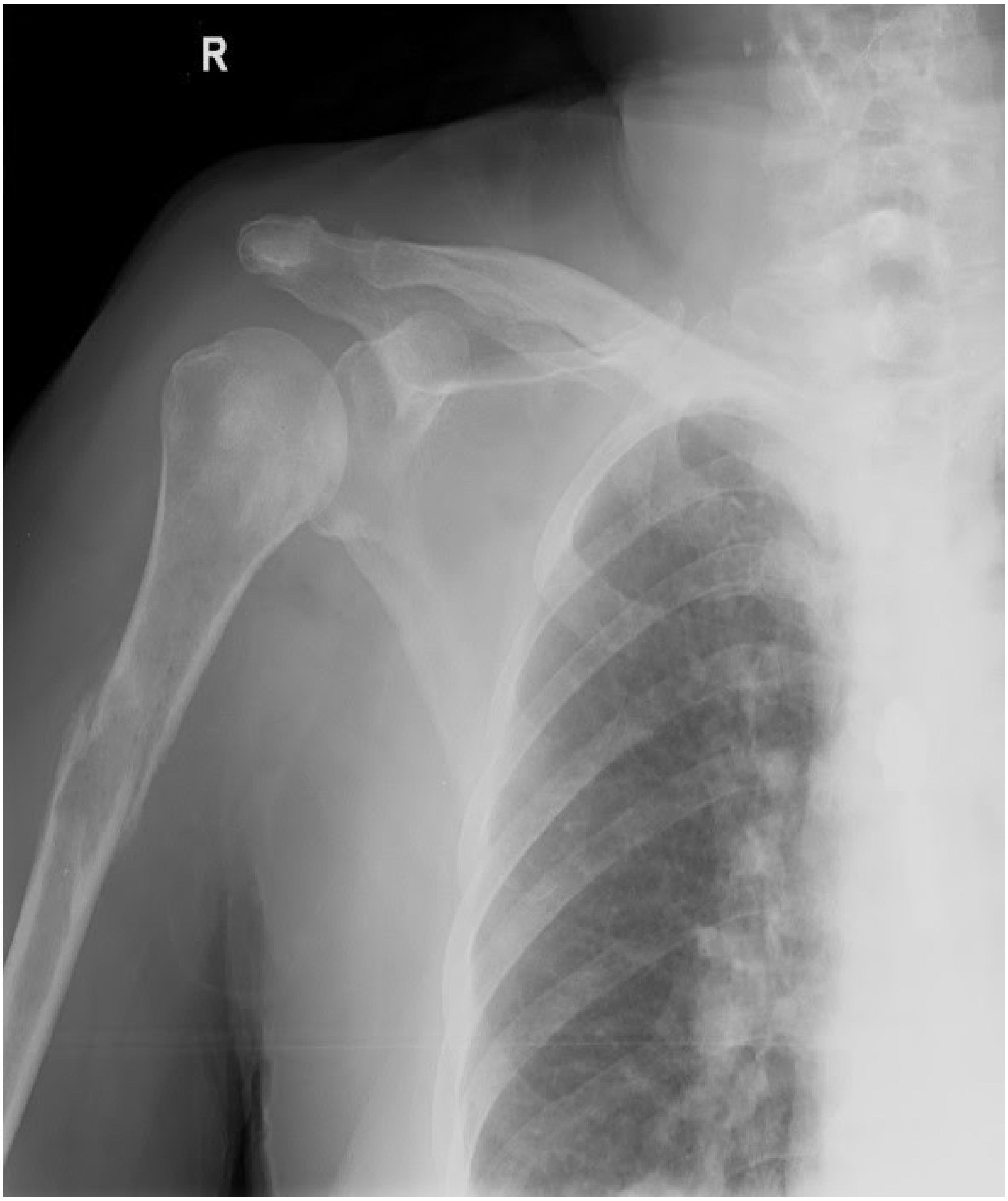
R

40. Jakie leczenie zastosujesz? *

Zaznacz tylko jedną odpowiedź.

- Gwóźdź śródszpikowy z resekcją guza z zastosowaniem cementu kostnego
- Gwóźdź śródszpikowy bez resekcji guza
- Płyta i śruby
- Endoproteza modularna trzonu kości z wycięciem zmiany
- Brak wskazań do leczenia operacyjnego
- Inne: ____________________

Przypadek 14

Pacjentka 60 r.ż.
- złamanie patologiczne
- rak piersi ze zmianą przerzutową do trzonu kości ramiennej (potwierdzony w badaniu histopatologicznym)
- silne dolegliwości bólowe, powodujące niepełnosprawność
- przewidywana długość życia poniżej 6 miesięcy

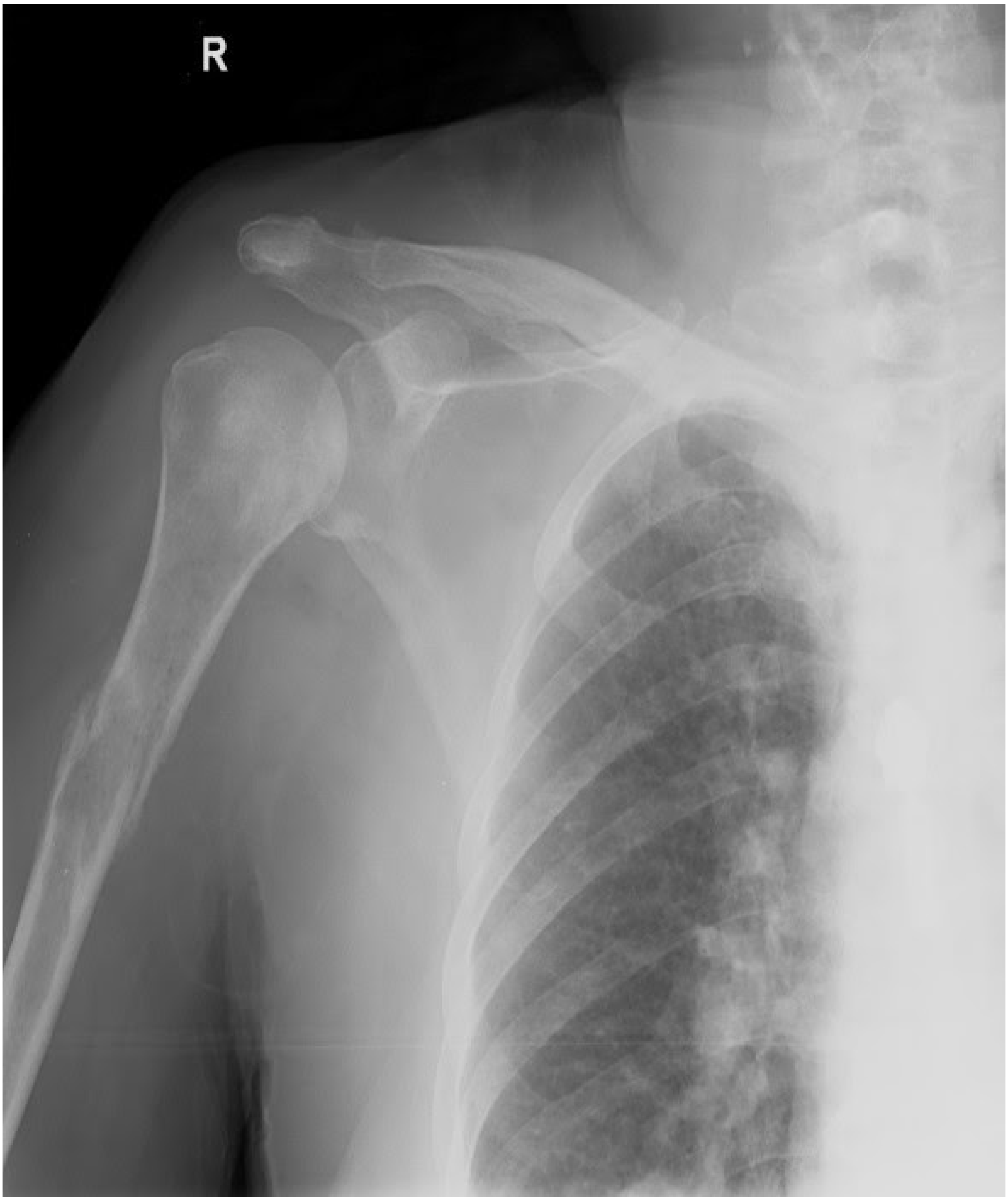
R

41. Jakie leczenie zastosujesz? *

Zaznacz tylko jedną odpowiedź.

- Gwóźdź śródszpikowy z resekcją guza z zastosowaniem cementu kostnego
- Gwóźdź śródszpikowy bez resekcji guza
- Płyta i śruby
- Endoproteza modularna trzonu kości z wycięciem zmiany
- Brak wskazań do leczenia operacyjnego
- Inne: ____________________

Przypadek 15

Pacjentka 60 r.ż.
- bez cech złamania patologicznego na RTG, wysokie ryzyko złamania patologicznego
- rak piersi ze zmianą przerzutową do trzonu kości udowej (potwierdzony w badaniu histopatologicznym)
- silne dolegliwości bólowe, powodujące niepełnosprawność
- przewidywana długość życia powyżej 12 miesięcy

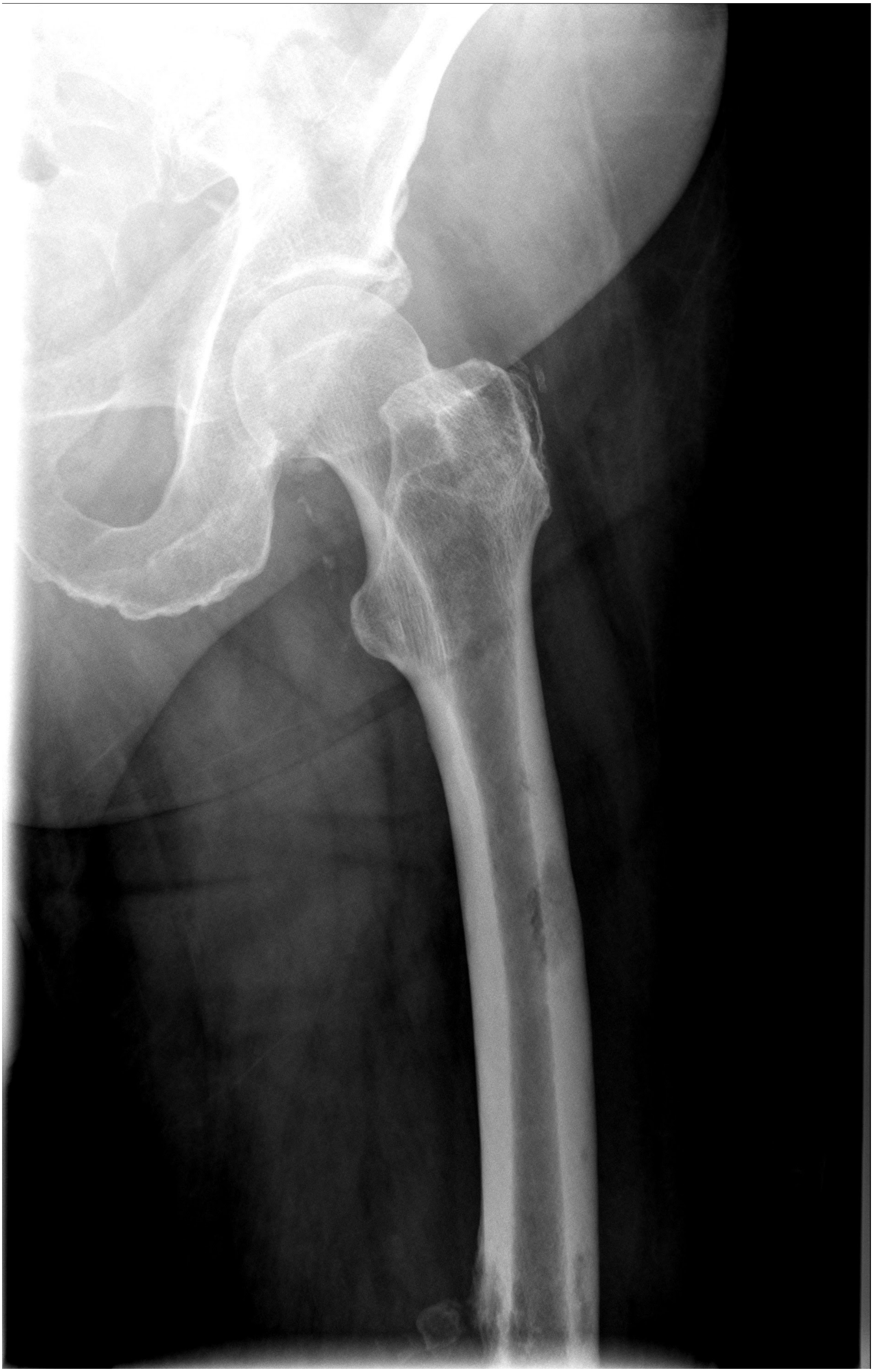

42. Jakie leczenie zastosujesz? *

Zaznacz tylko jedną odpowiedź.

- Gwóźdź śródszpikowy z resekcją guza z zastosowaniem cementu kostnego
- Gwóźdź śródszpikowy bez resekcji guza
- Płyta i śruby
- Endoproteza modularna trzonu kości z wycięciem zmiany
- Brak wskazań do leczenia operacyjnego
- Inne: ______________________

Przypadek 16

Pacjentka 60 r.ż.
- bez cech złamania patologicznego na RTG, wysokie ryzyko złamania patologicznego
- rak piersi ze zmianą przerzutową do trzonu kości udowej (potwierdzony w badaniu histopatologicznym)
- silne dolegliwości bólowe, powodujące niepełnosprawność
- przewidywana długość życia poniżej 6 miesięcy

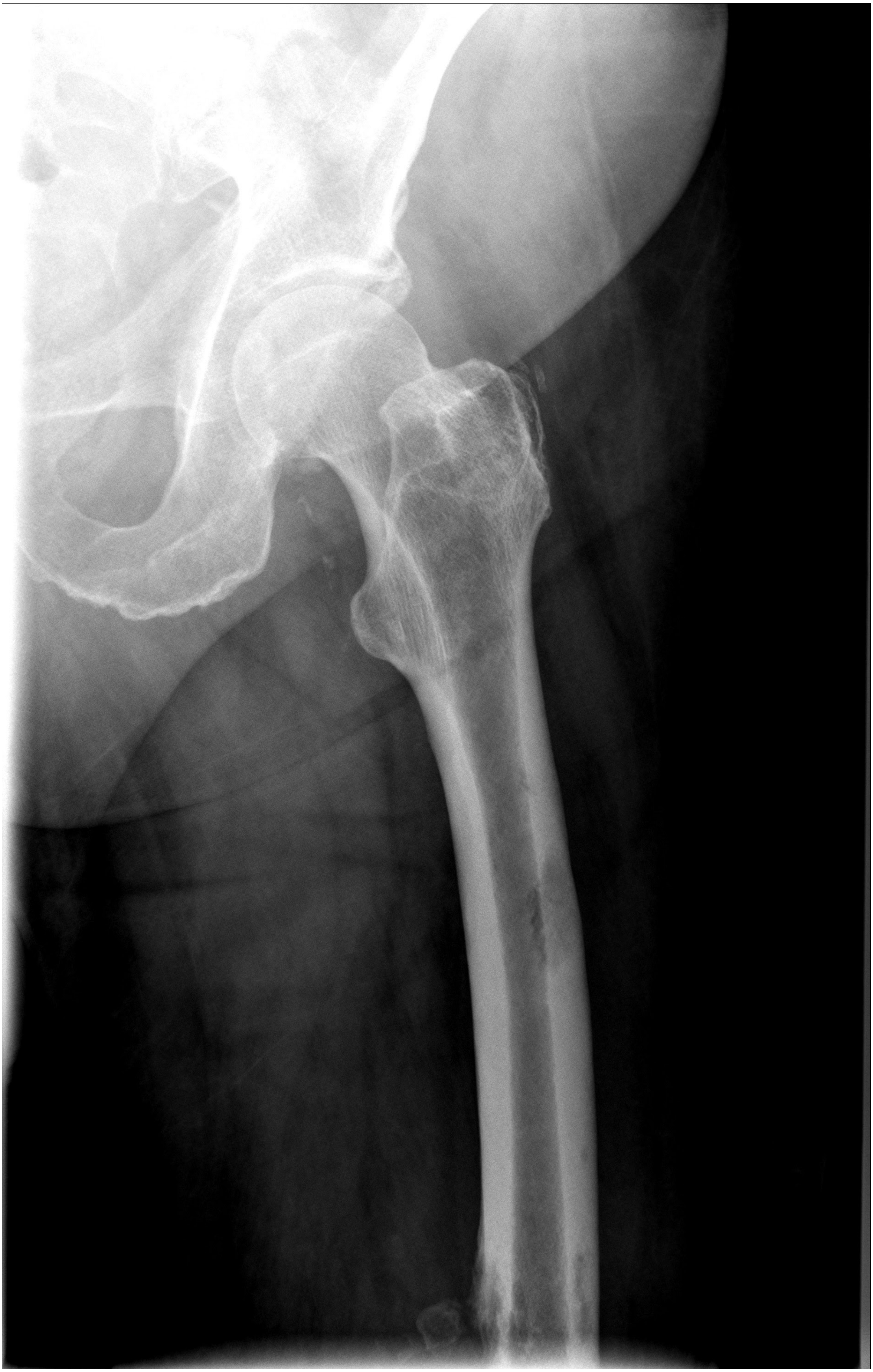

43. Jakie leczenie zastosujesz? *

Zaznacz tylko jedną odpowiedź.

- Gwóźdź śródszpikowy z resekcją guza z zastosowaniem cementu kostnego
- Gwóźdź śródszpikowy bez resekcji guza
- Płyta i śruby
- Endoproteza modularna trzonu kości z wycięciem zmiany
- Brak wskazań do leczenia operacyjnego
- Inne: ____________________

44. W przypadku pacjentki z pojedynczą zmianą przerzutową raka nerki do trzonu kości udowej, wykrytą przypadkowo (bez dolegliwości bólowych) z dobrym rokowaniem i odstępem >3 lat od wykrycia zmiany pierwotnej, jaką metodę postępowania preferowałbyś/preferowałabyś po wykluczeniu pierwotnego nowotworu kości? *

Zaznacz tylko jedną odpowiedź.

- Brak wskazań do leczenia operacyjnego
- Gwóźdź śródszpikowy z resekcją guza z zastosowaniem cementu kostnego
- Gwóźdź śródszpikowy bez resekcji guza
- Płyta i śruby
- Endoproteza modularna trzonu kości z wycięciem zmiany
- Inne: ____________________

Uwagi końcowe

45. Dziękujemy bardzo za wypełnienie ankiety. Jeśli mają Państwo jakieś uwagi, pytania lub chcieliby Państwo podzielić się bardziej szczegółowo swoim doświadczeniem w postępowaniu ze zmianami przerzutowymi do kości, to zapraszamy do wypełnienia poniższego pola:

Zdjęcia radiologiczne - źródła

Przypadek 1-2) Case courtesy of Dr Bruno Di Muzio, Radiopaedia.org. From the case rID: 26536
Przypadek 3-4) Case courtesy of Dr Subash Thapa, Radiopaedia.org. From the case rID: 40234
Przypadek 5-6) Case courtesy of Dr Sajoscha Sorrentino, Radiopaedia.org. From the case rID: 16538
Przypadek 7-8) Case courtesy of Dr Ian Bickle, Radiopaedia.org. From the case rID: 75119
Przypadek 9-10) Case courtesy of Assoc Prof Frank Gaillard, Radiopaedia.org. From the case rID: 23325
Przypadek 11-12) Case courtesy of Dr Henry Knipe, Radiopaedia.org. From the case rID: 30462
Przypadek 13-14) Case courtesy of Dr Henry Knipe, Radiopaedia.org. From the case rID: 47200
Przypadek 15-16) Case courtesy of Dr Lawrence Oh, Radiopaedia.org. From the case rID: 28869

Ta treść nie została utworzona ani zatwierdzona przez Google.

Formularze Google
