# Supplementary material for: Trends in Diagnosis and Surgical Treatment of Bone Metastases among Orthopedic Surgeons
Source: J Clin Med. 2022 Jul 23;11(15):4284. doi: 10.3390/jcm11154284 (PMC9341391; doi:10.3390/jcm11154284)
Supplement: Supplementary file 1 [file jcm-11-04284-s001.zip › jcm-1757820-supplementary/File S2 - Survey ENG.pdf]

Trends in diagnostic and therapeutic management in patients with metastases to the bones - a survey among orthopedic surgeons in Poland

Dear Colleagues,

This is an anonymous survey to examine the current trends in the diagnosis, qualification and treatment of patients with bone metastases. It is divided into 2 main parts - theoretical part and clinical cases.

It should take up to 15 minutes to complete all of the questions.

The questionnaire was created as a research project carried out at the Department and Clinic of Orthopedics and Traumatology of the Medical University of Gdańsk (Poland).

Thank you in advance for your time and for completing the survey below.

I am aware that participation in the survey is voluntary and anonymous and I agree to participate in the survey by going to the next part of the survey

* Required

Basic information - group characteristics

1. What is your gender?

Mark only one oval.

- ⬭ Male
- ⬭ Female

2. How many years have you been in practice as an orthopedic surgeon? *

Mark only one oval.

- 0-5
- 6-10
- 11-20
- >20

3. In which kind of institution do you work currently? *

Mark only one oval.

- University Hospital/Tertiary referral hospital
- District Hospital
- Private medical practice
- Other: ______________________

4. How often do you performe surgery for bone metastases (average number over a year)? *

Mark only one oval.

- 0-5
- 6-10
- 11-20
- >20

5. Main field of interests: (more than 1 answer is possible - max. 3) *

Check all that apply.

- [ ] General orthopedics
- [ ] Traumatology
- [ ] Musculoskeletal oncology
- [ ] Joints arthroplasty
- [ ] Arthroscopy and minimal invasive orthopedics
- [ ] Spine surgery
- [ ] Pediatric orthopedics
- [ ] Hand surgery
- [ ] Foot surgery

Other: [ ] ______________________

6. How confident do you feel when performing surgery on metastatic bone tumors? *

Mark only one oval.

	1	2	3	4	5	
Definitely unconfident	( )	( )	( )	( )	( )	Definitely confident

Diagnostic path and qualification for surgery

7. What is your preferred management of a patient who comes to the emergency room / orthopedic clinic with a suspicion of bone metastasis? *

Mark only one oval.

( ) Referral to an oncology clinic
( ) Referral to an oncological orthopedic clinic
( ) Admission to the ward and qualification for biopsy and further treatment
( ) Other: ______________________

8. What scales do you use to qualify a patient for the surgery (you can choose more than one answer)? *

Check all that apply.

- [ ] Bollen Prognostic Scale
- [ ] The Modified Bauer Score
- [ ] Forsberga (Bayesian-Estimated Tools for Survival - BETS)
- [ ] Katagiri Score
- [ ] Mirels Classification
- [ ] Karnofsky Scale
- [ ] Musculoskeletal Tumor Society Scoring System (MSTS)
- [ ] Capanna Classification
- [ ] I don't use scales at all

Other: [ ] ______________________

9. Are you using in your practise the PATHFx app to assess the life expectancy of patients with bone metastases (www.pathfx.org)? *

Mark only one oval.

- ( ) Yes, I use this app in my practise
- ( ) No, I know this app, but I don't use it in my practise
- ( ) No, I don't know this app and I don't use it in my practise

10. What type of biopsy do you prefer when bone metastasis is suspected? *

Mark only one oval.

- ( ) Core needle biopsy
- ( ) Trepanobiopsy
- ( ) Open biopsy
- ( ) I never perform a biopsy if I suspect a bone metastasis
- ( ) Other: ______________________

11. How much do you agree with the statement below - Patients with a single lesion suspected of having bone metastasis should have a biopsy to rule out primary bone tumors? *

Mark only one oval.

	1	2	3	4	5	
Strongly disagree	◯	◯	◯	◯	◯	Strongly agree

12. What imaging methods do you use in your practice to qualify a patient for surgical treatment of a metastatic bone tumor? (you can choose more than one answer) *

Check all that apply.

- [ ] X-ray
- [ ] Computed tomography (CT)
- [ ] Magnetic resonance imaging (MRI)
- [ ] Bone scan
- [ ] PET-CT

Other: [ ] ____________________

How important are the following symptoms /factors to you in qualifying a patient for surgery for bone metastases?

13. Level of pain *

Mark only one oval.

	1	2	3	4	5	
Definitely unimportant	◯	◯	◯	◯	◯	Definitely important

14. The number of bone metastases (single / multiple) *

Mark only one oval.

	1	2	3	4	5	
Definitely unimportant	◯	◯	◯	◯	◯	Definitely important

15. The size of the lesion and the level of bone destruction *

Mark only one oval.

	1	2	3	4	5	
Definitely unimportant	◯	◯	◯	◯	◯	Definitely important

16. The occurrence of pathological fracture *

Mark only one oval.

	1	2	3	4	5	
Definitely unimportant	◯	◯	◯	◯	◯	Definitely important

17. Impending fracture (hight risk of pathological fracture) *

Mark only one oval.

	1	2	3	4	5	
Definitely unimportant	◯	◯	◯	◯	◯	Definitely important

18. Patient life expectancy *

Mark only one oval.

	1	2	3	4	5	
Definitely unimportant						Definitely important

19. The type of primary tumor *

Mark only one oval.

	1	2	3	4	5	
Definitely unimportant						Definitely important

20. The presence of metastatic changes in visceral organs (visceral metastases) *

Mark only one oval.

	1	2	3	4	5	
Definitely unimportant						Definitely important

21. Functional assessment and patient mobility *

Mark only one oval.

	1	2	3	4	5	
Definitely unimportant						Definitely important

22. The quality of the patient's life *

Mark only one oval.

	1	2	3	4	5	
Definitely unimportant	⬭	⬭	⬭	⬭	⬭	Definitely important

23. Time from the diagnosis of the primary tumor to the detection of a metastatic lesion *

Mark only one oval.

	1	2	3	4	5	
Definitely unimportant	⬭	⬭	⬭	⬭	⬭	Definitely important

24. Hemoglobin concentration in blood *

Mark only one oval.

	1	2	3	4	5	
Definitely unimportant	⬭	⬭	⬭	⬭	⬭	Definitely important

Treatment

25. Do you use / recommend before the surgery of bone metastases of kidney or thyroid cancer blood vessel embolization of the metastatic tumor, with its rich vascularization? *

Mark only one oval.

	1	2	3	4	5	
Never	⬭	⬭	⬭	⬭	⬭	Always

26. Where do you refer the patient after the surgical treatment of bone metastatic tumors? *

Mark only one oval.

- Oncology clinic
- General Practice doctor (GP)
- Orthopedic outpatients clinic
- Other: ____________________

27. What method of bone filling do you use in the surgical treatment of bone metastases? *

Check all that apply.

- [ ] Polymethyl methacrylate (PMMA)
- [ ] Fresh-frozen bone allograft
- [ ] Autogenous iliac bone graft
- [ ] Vascularized tibia bone graft

Other: [ ] ____________________

Clinical cases

In this section, please read the examples of clinical cases and choose the treatment method that you would use in your practice.

The presented photos are for reference only. Please follow the information provided in the text mainly when choosing a treatment. Cases may differ only in individual distractors (mainly 'life expectancy'), so please read the description carefully.

Case 1

A 60-year-old patient
- pathological fracture
- kidney cancer with metastases to the femoral shaft (confirmed by histopathology)
- severe pain causing disability
- life expectancy over 12 months

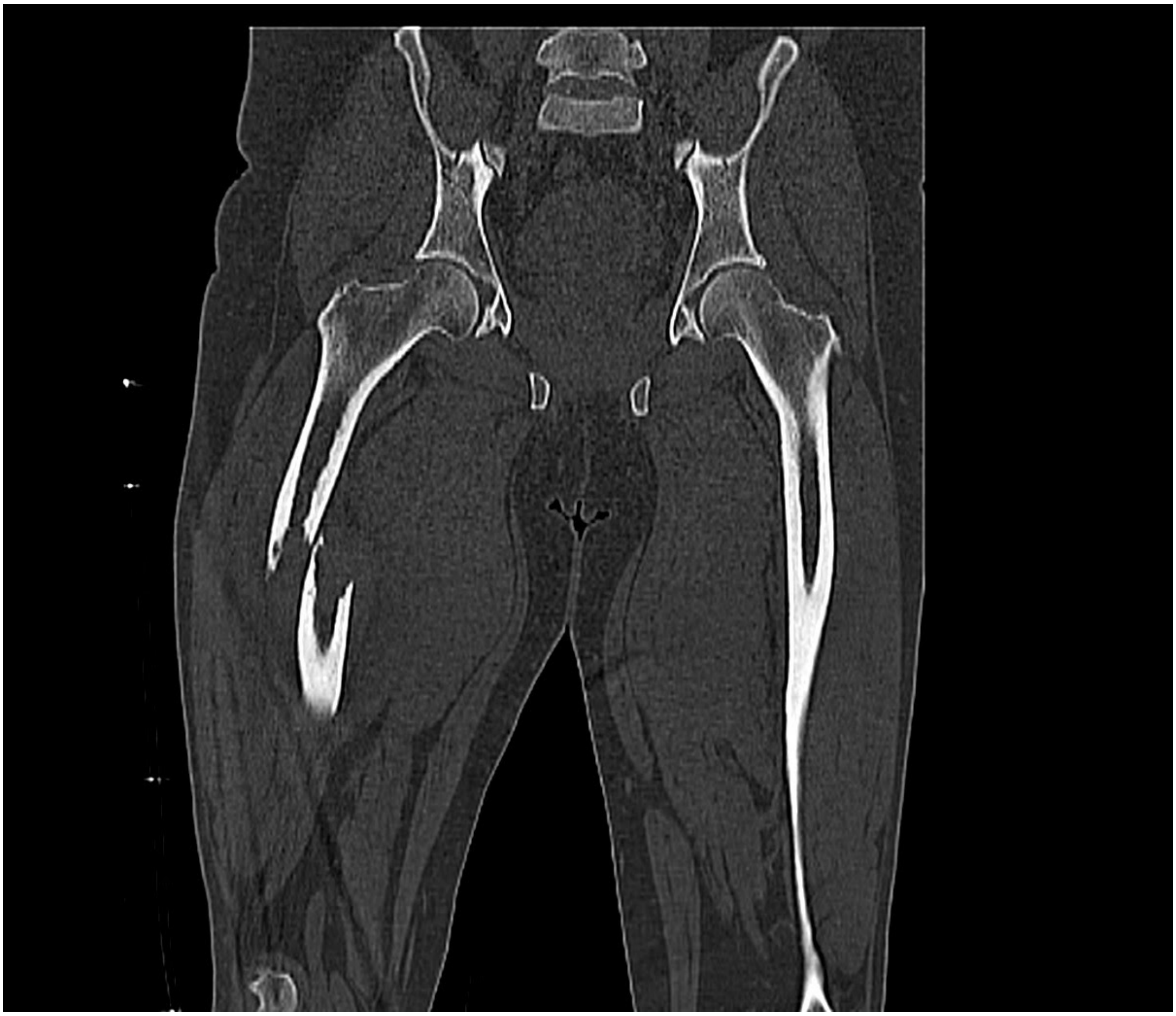

28. Which treatment method do you prefer? *

Mark only one oval.

- Intramedullary nail with tumor resection and using Polymethyl methacrylate (PMMA)
- Intramedullary nail without tumor resection
- Plate-screw fixation device
- Modular endoprosthesis with tumor resection
- No indications for surgical treatment
- Other: ______________________

Case 2

A 60-year-old patient
- pathological fracture
- kidney cancer with metastases to the femoral shaft (confirmed by histopathology)
- severe pain causing disability
- life expectancy less than 6 months

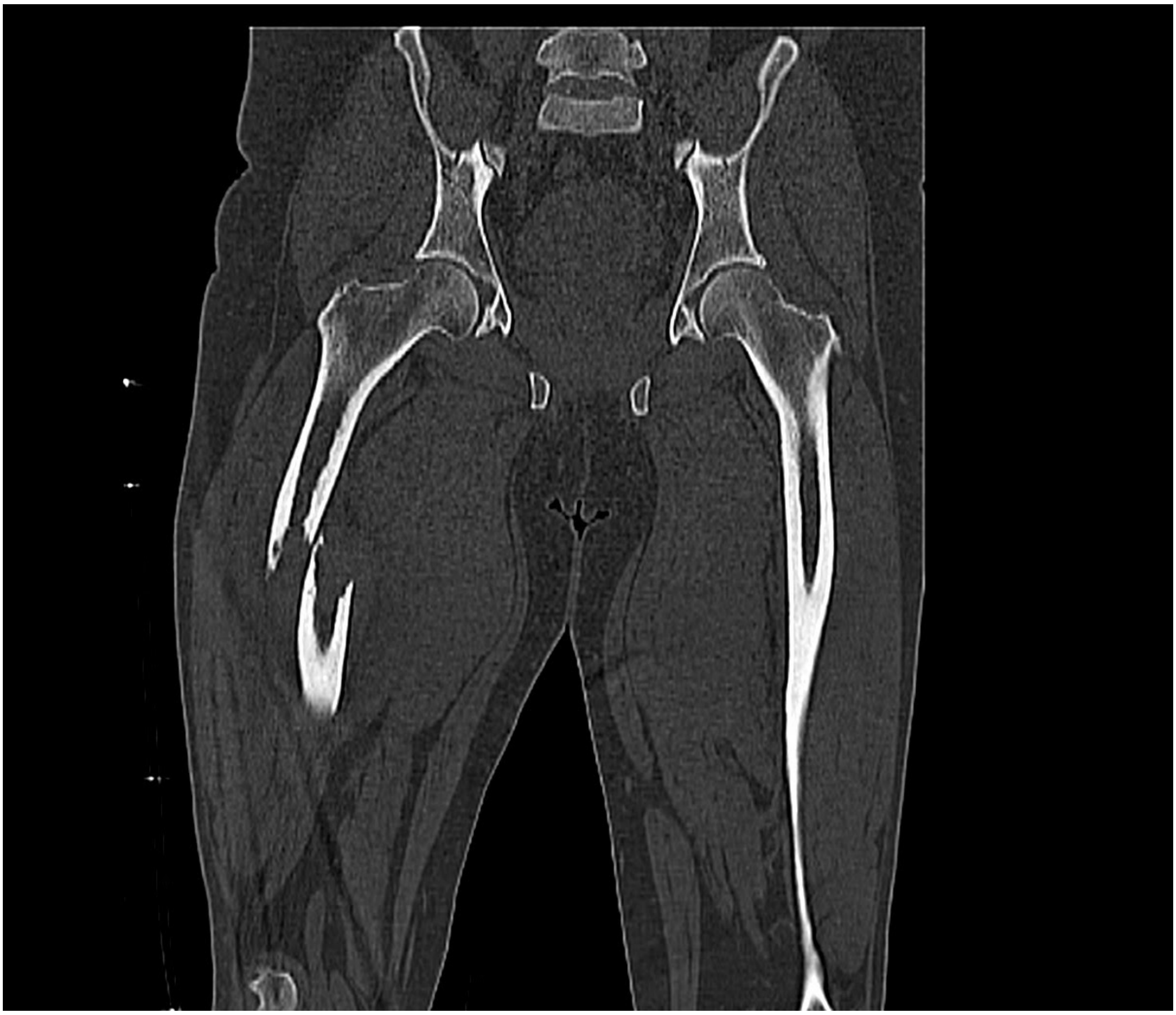

29. Which treatment method do you prefer? *

Mark only one oval.

- Intramedullary nail with tumor resection and using Polymethyl methacrylate (PMMA)
- Intramedullary nail without tumor resection
- Plate-screw fixation device
- Modular endoprosthesis with tumor resection
- No indications for surgical treatment
- Other: ______________________

Case 3

A 60-year-old patient
- no evidence of pathological fracture on X-ray, high risk of pathological fracture (impending fracture)
- kidney cancer with metastases to the shaft of the humerus (confirmed by histopathology)
- severe pain causing disability
- life expectancy over 12 months

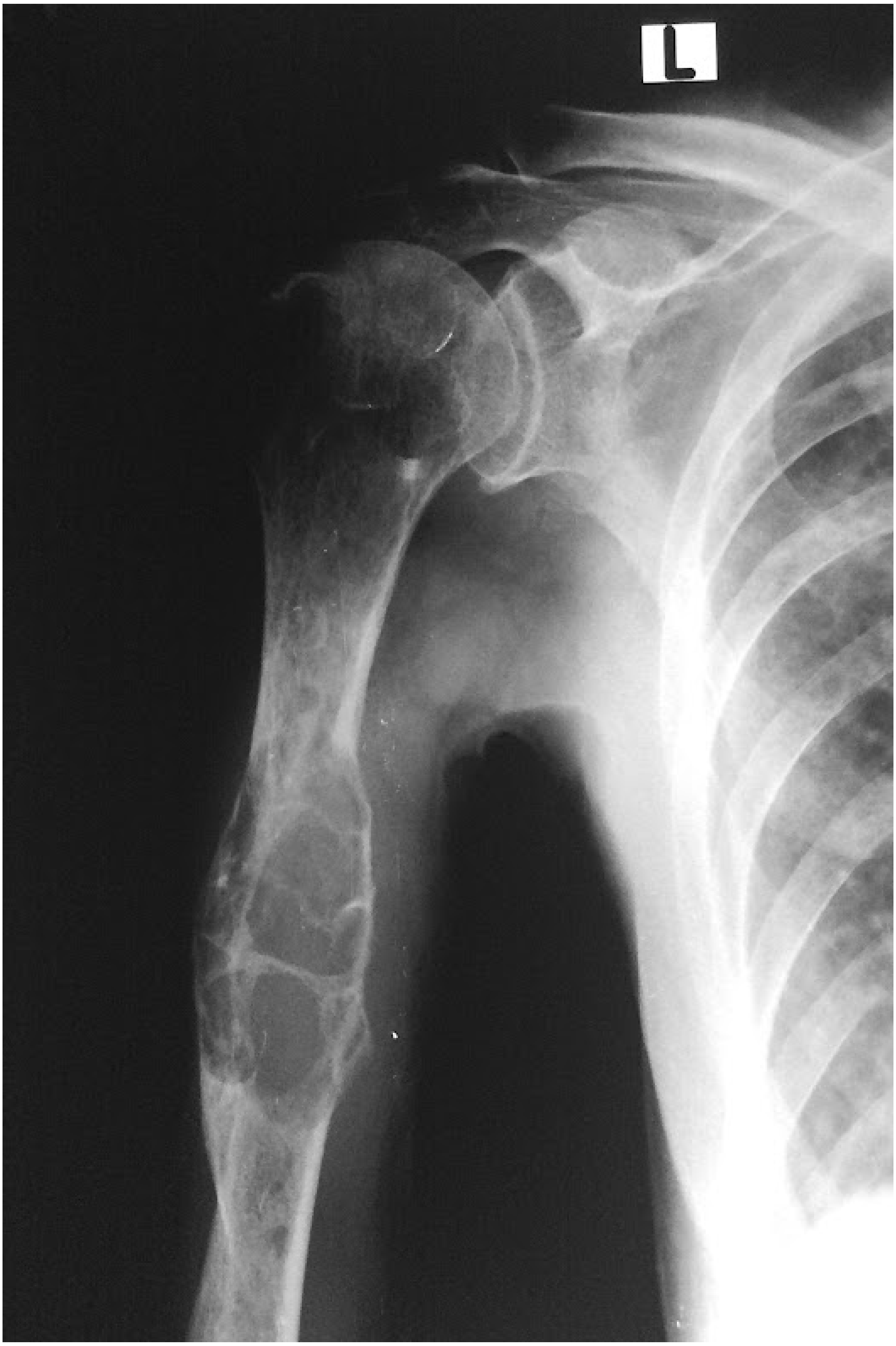
L

30. Which treatment method do you prefer? *

Mark only one oval.

- Intramedullary nail with tumor resection and using Polymethyl methacrylate (PMMA)
- Intramedullary nail without tumor resection
- Plate-screw fixation device
- Modular endoprosthesis with tumor resection
- No indications for surgical treatment
- Other: ______________

Case 4

A 60-year-old patient
- no evidence of pathological fracture on X-ray, high risk of pathological fracture (impending fracture)
- kidney cancer with metastases to the shaft of the humerus (confirmed by histopathology)
- severe pain causing disability
- life expectancy less than 6 months

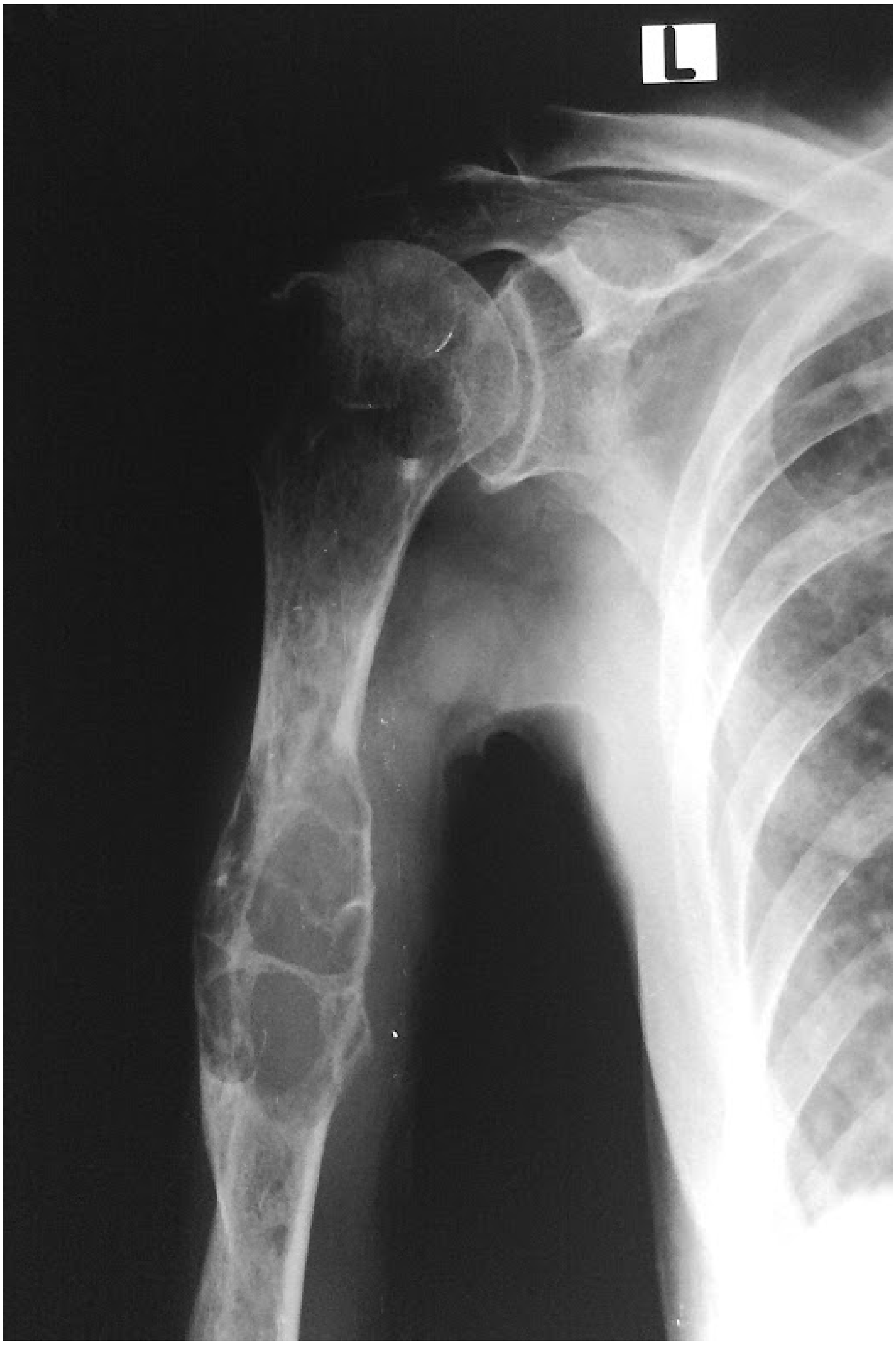
L

31. Which treatment method do you prefer? *

Mark only one oval.

- Intramedullary nail with tumor resection and using Polymethyl methacrylate (PMMA)
- Intramedullary nail without tumor resection
- Plate-screw fixation device
- Modular endoprosthesis with tumor resection
- No indications for surgical treatment
- Other: ______________________

Case 5

A 60-year-old patient
- pathological fracture
- breast cancer with metastases to the femoral shaft (confirmed by histopathology)
- severe pain causing disability
- life expectancy over 12 months

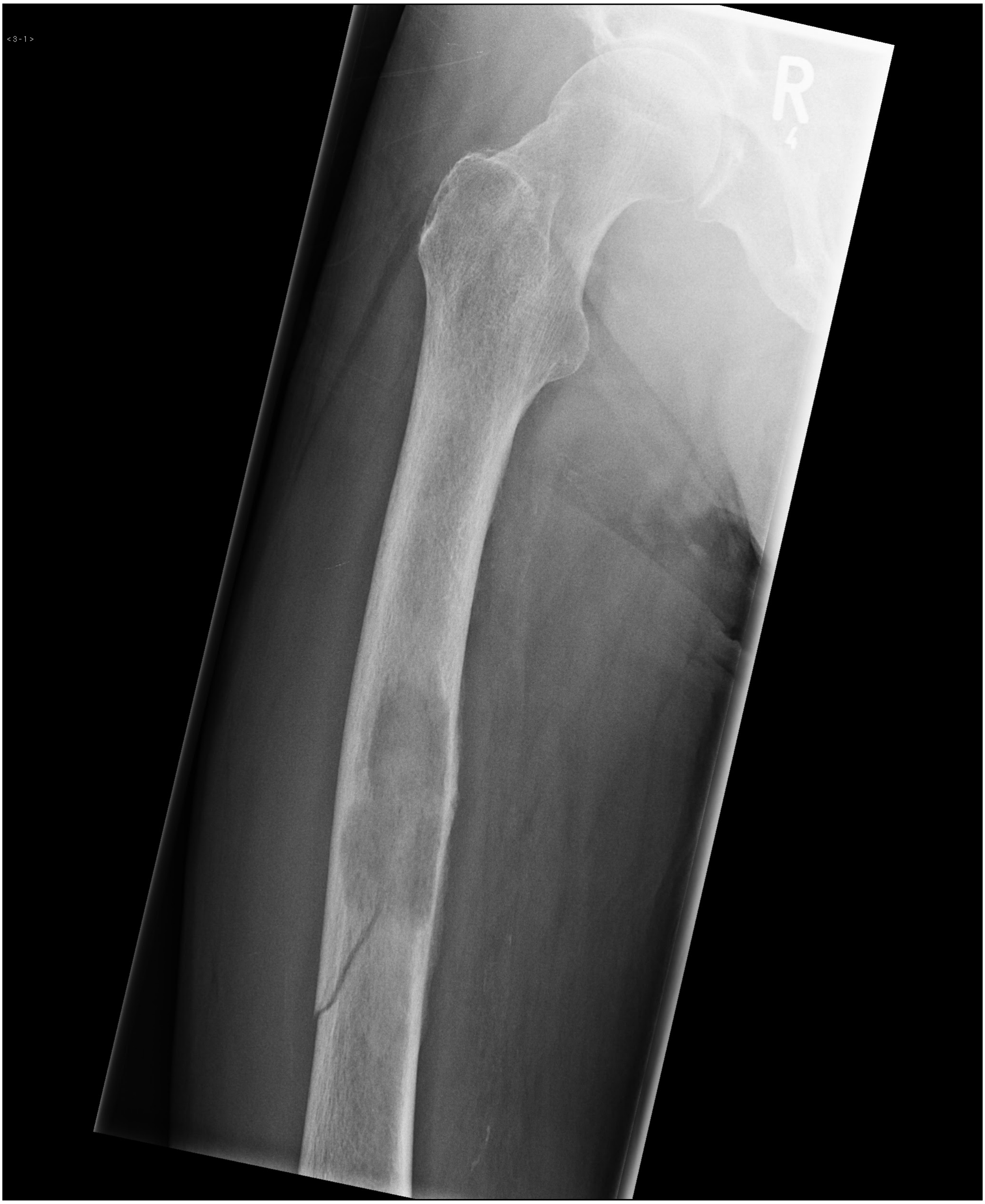
R

32. Which treatment method do you prefer? *

Mark only one oval.

- Intramedullary nail with tumor resection and using Polymethyl methacrylate (PMMA)
- Intramedullary nail without tumor resection
- Plate-screw fixation device
- Modular endoprosthesis with tumor resection
- No indications for surgical treatment
- Other: ____________________

Case 6

A 60-year-old patient
- pathological fracture
- breast cancer with metastases to the femoral shaft (confirmed by histopathology)
- severe pain causing disability
- life expectancy less than 6 months

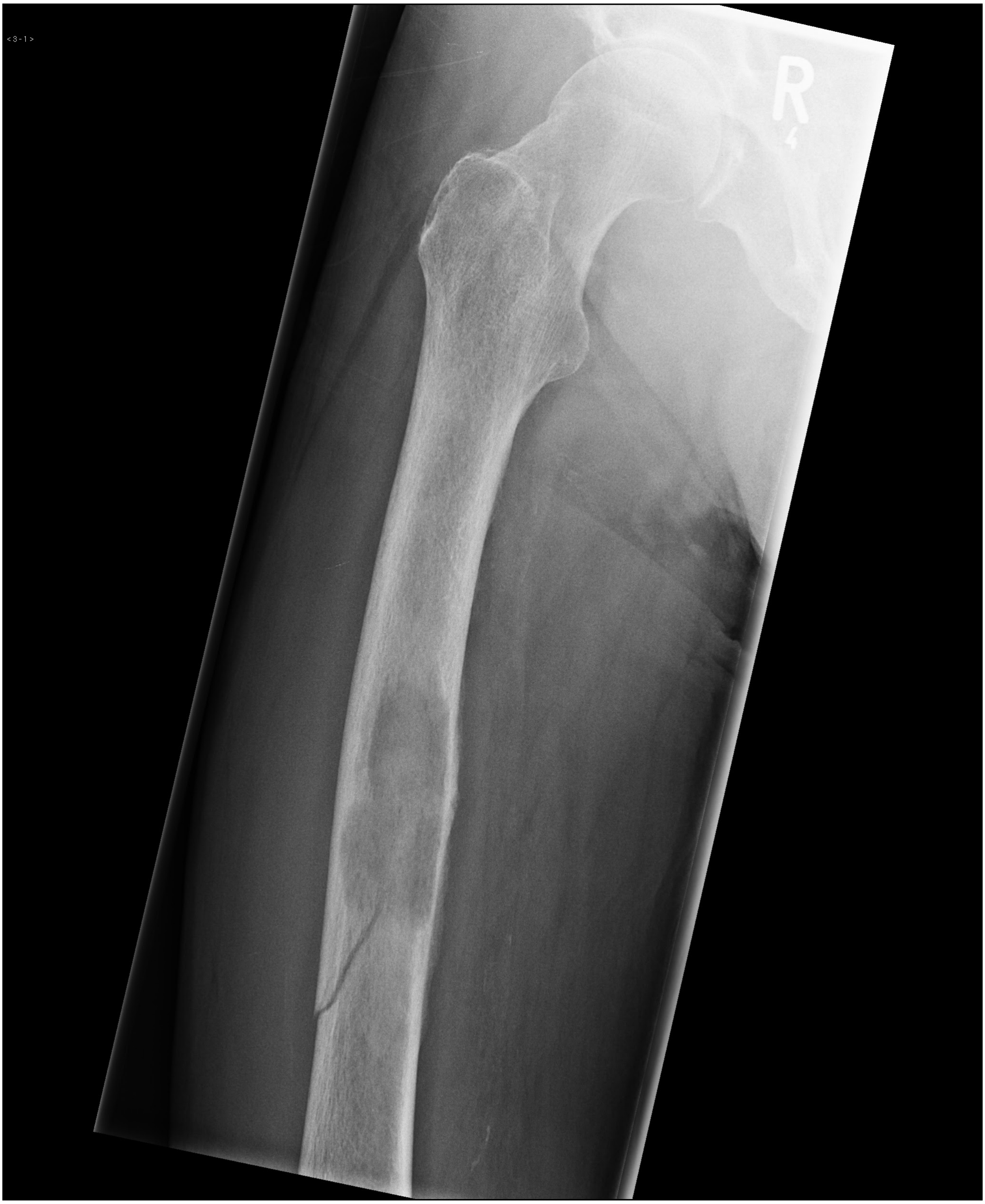
R

33. Which treatment method do you prefer? *

Mark only one oval.

- Intramedullary nail with tumor resection and using Polymethyl methacrylate (PMMA)
- Intramedullary nail without tumor resection
- Plate-screw fixation device
- Modular endoprosthesis with tumor resection
- No indications for surgical treatment
- Other: ____________________

Case 7

A 60-year-old patient
- pathological fracture
- kidney cancer with metastases to the shaft of the humerus (confirmed by histopathology)
- severe pain causing disability
- life expectancy over 12 months

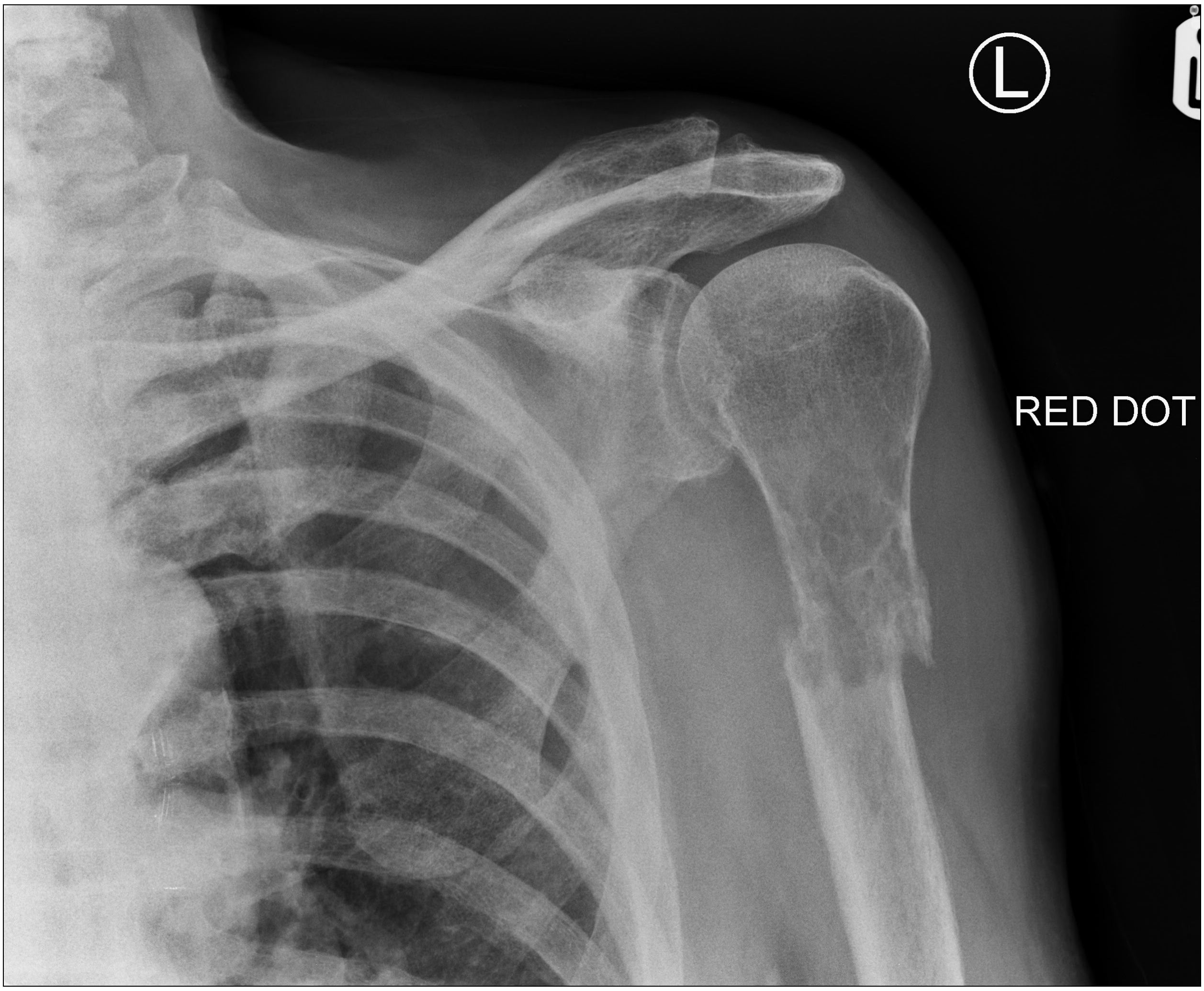

34. Which treatment method do you prefer? *

Mark only one oval.

- Intramedullary nail with tumor resection and using Polymethyl methacrylate (PMMA)
- Intramedullary nail without tumor resection
- Plate-screw fixation device
- Modular endoprosthesis with tumor resection
- No indications for surgical treatment
- Other: ______________________

Case 8

A 60-year-old patient
- pathological fracture
- kidney cancer with metastases to the shaft of the humerus (confirmed by histopathology)
- severe pain causing disability
- life expectancy less than 6 months

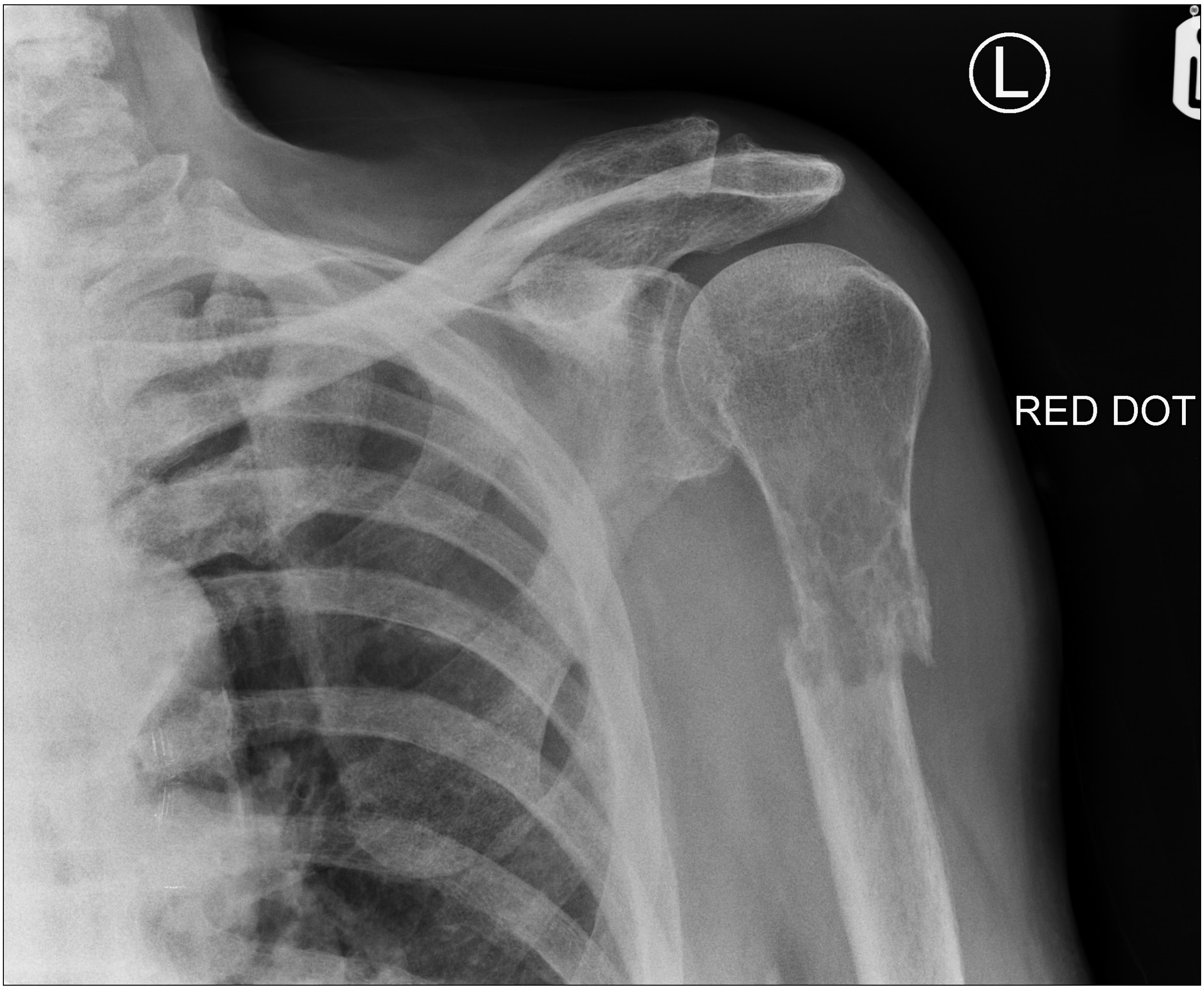

35. Which treatment method do you prefer? *

Mark only one oval.

- Intramedullary nail with tumor resection and using Polymethyl methacrylate (PMMA)
- Intramedullary nail without tumor resection
- Plate-screw fixation device
- Modular endoprosthesis with tumor resection
- No indications for surgical treatment
- Other: ____________________

Case 9

A 60-year-old patient
- no evidence of pathological fracture on X-ray, high risk of pathological fracture (impending fracture)
- kidney cancer with metastases to the femoral shaft (confirmed by histopathology)
- severe pain causing disability
- life expectancy over 12 months

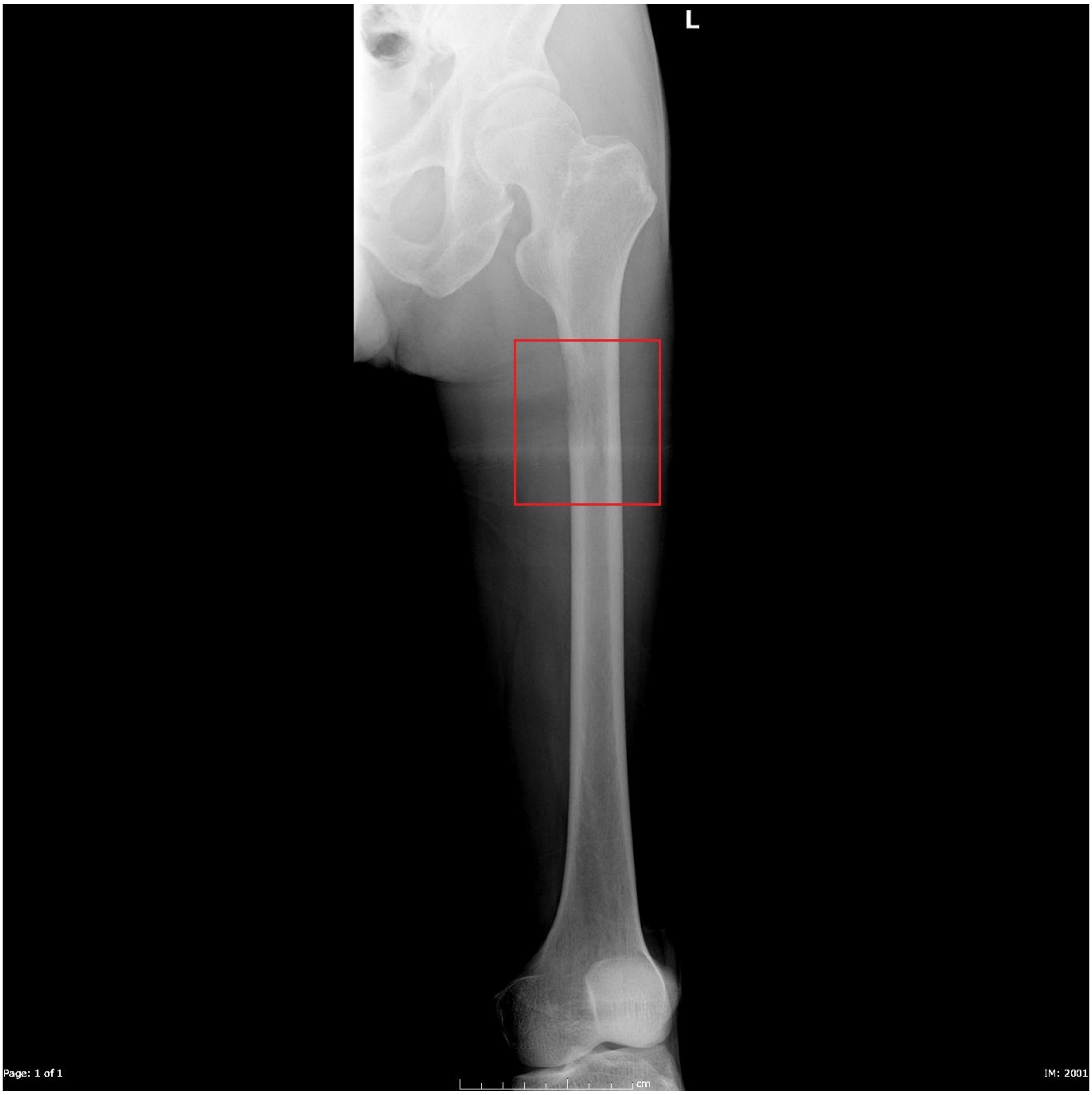

36. Which treatment method do you prefer? *

Mark only one oval.

- Intramedullary nail with tumor resection and using Polymethyl methacrylate (PMMA)
- Intramedullary nail without tumor resection
- Plate-screw fixation device
- Modular endoprosthesis with tumor resection
- No indications for surgical treatment
- Other: __________

Case 10

A 60-year-old patient
- no evidence of pathological fracture on X-ray, high risk of pathological fracture (impending fracture)
- kidney cancer with metastases to the femoral shaft (confirmed by histopathology)
- severe pain causing disability
- life expectancy less than 6 months

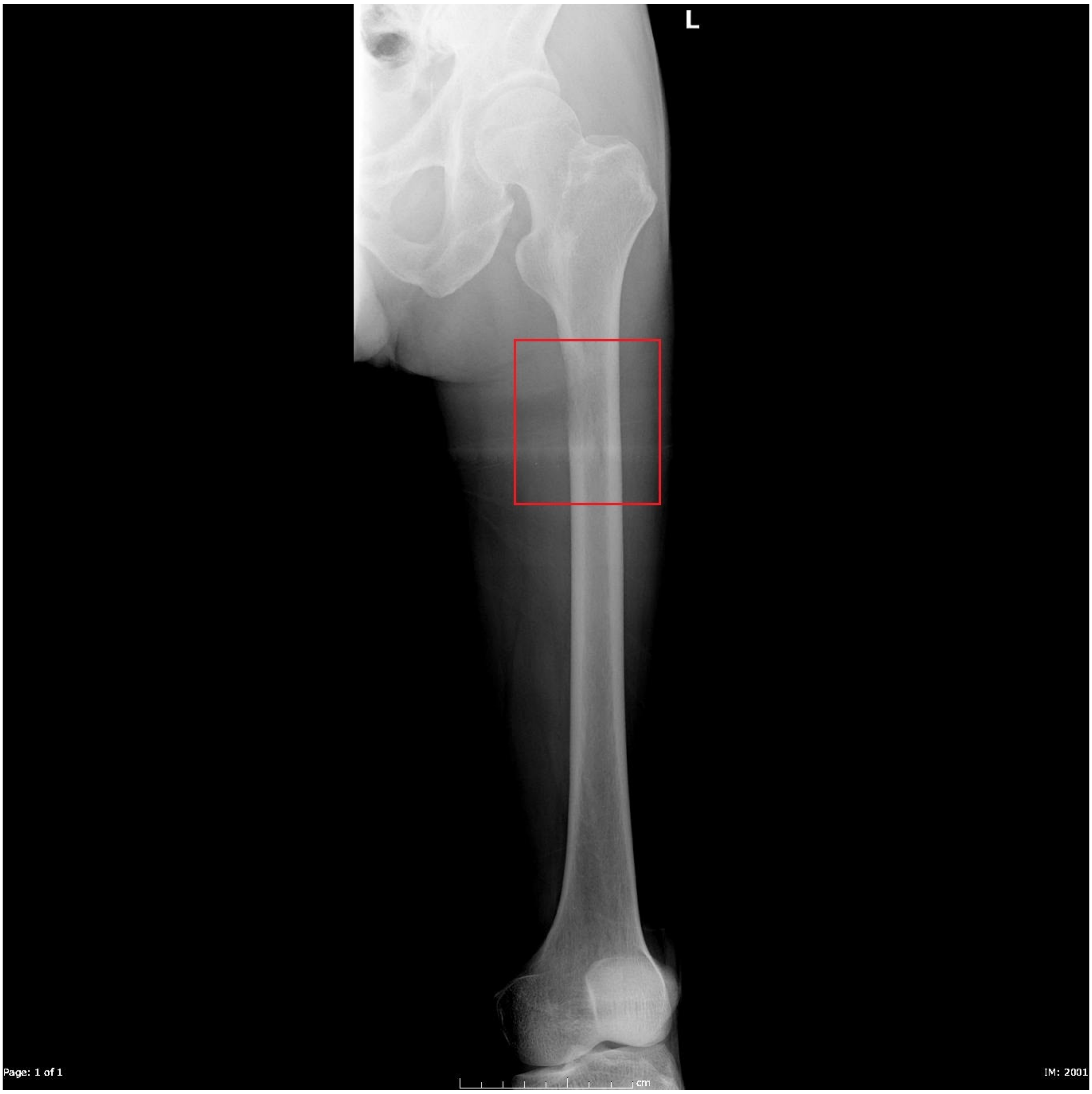

37. Which treatment method do you prefer? *

Mark only one oval.

- Intramedullary nail with tumor resection and using Polymethyl methacrylate (PMMA)
- Intramedullary nail without tumor resection
- Plate-screw fixation device
- Modular endoprosthesis with tumor resection
- No indications for surgical treatment
- Other: ______________________

Case 11

A 60-year-old patient
- no evidence of pathological fracture on X-ray, high risk of pathological fracture (impending fracture)
- breast cancer with metastases to the shaft of the humerus (confirmed by histopathology)
- severe pain causing disability
- life expectancy over 12 months

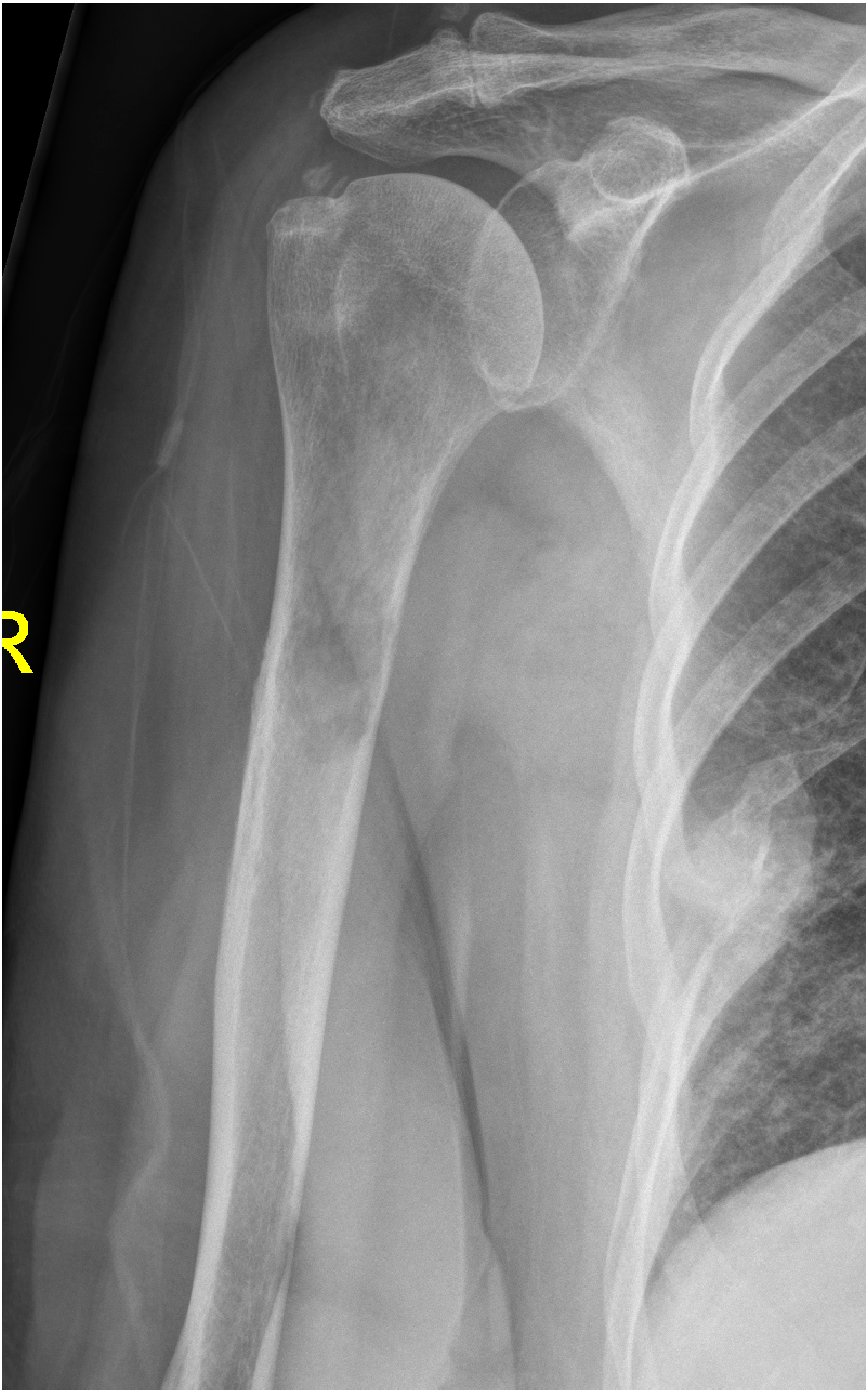
R

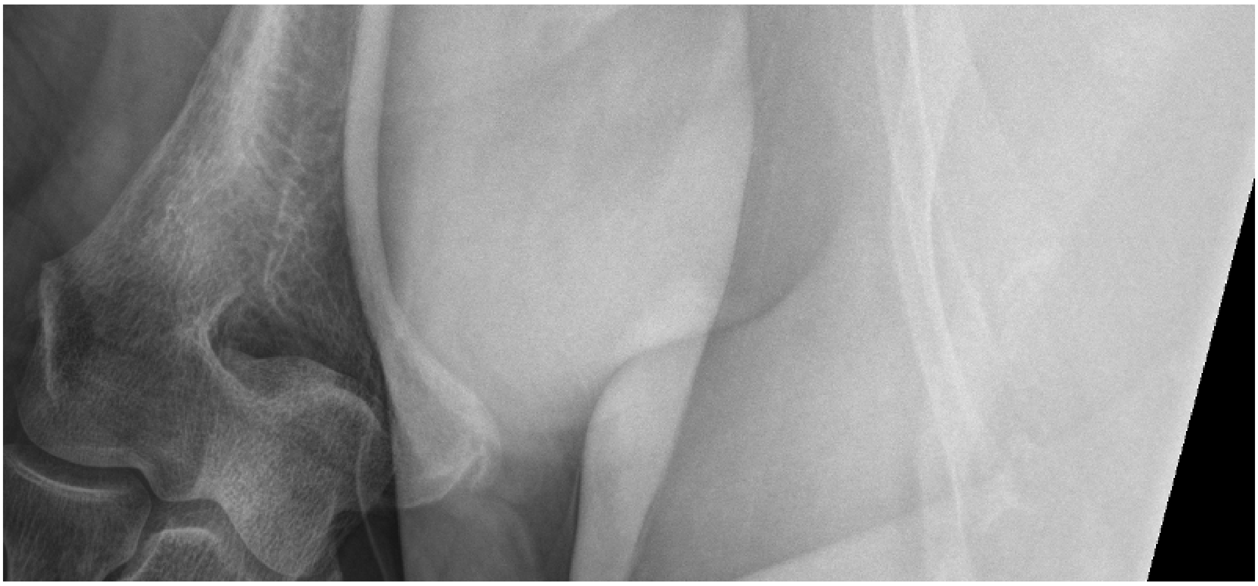

38. Which treatment method do you prefer? *

Mark only one oval.

- Intramedullary nail with tumor resection and using Polymethyl methacrylate (PMMA)
- Intramedullary nail without tumor resection
- Plate-screw fixation device
- Modular endoprosthesis with tumor resection
- No indications for surgical treatment
- Other: ____________________

Case 12

A 60-year-old patient
- no evidence of pathological fracture on X-ray, high risk of pathological fracture (impending fracture)
- breast cancer with metastases to the shaft of the humerus (confirmed by histopathology)
- severe pain causing disability
- life expectancy less than 6 months

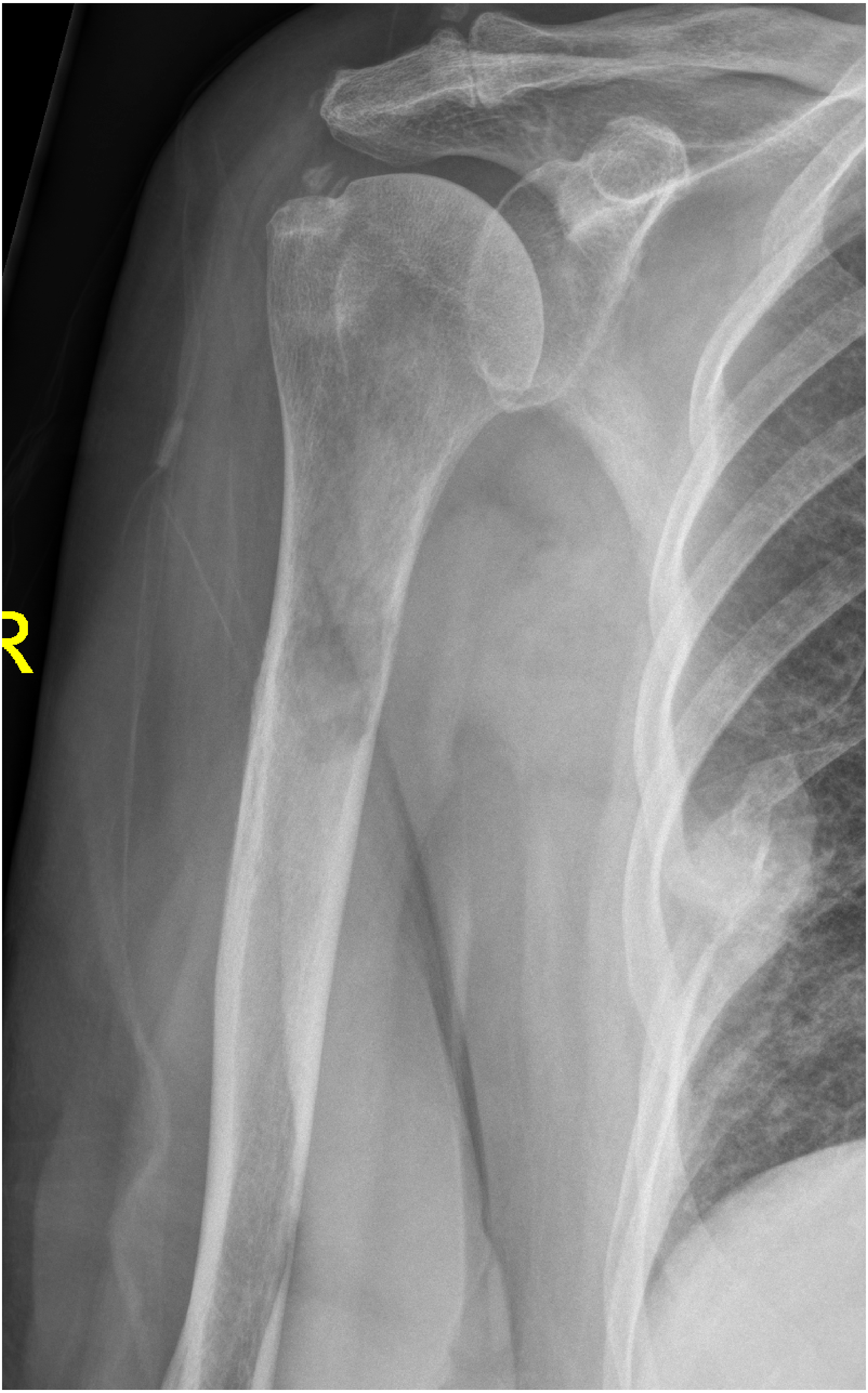
R

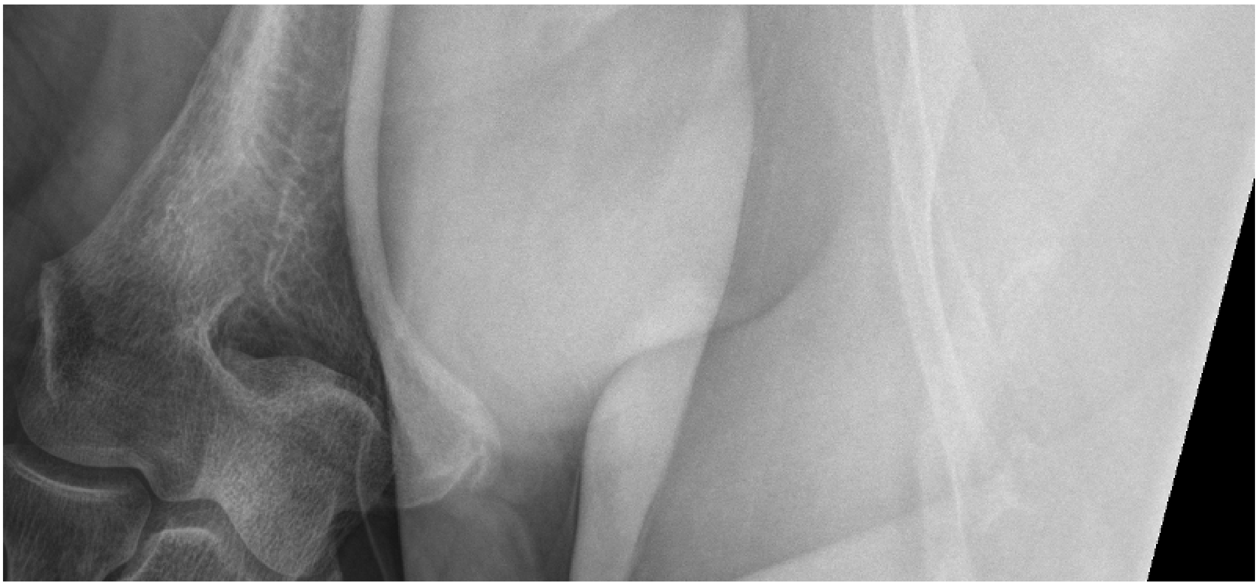

39. Which treatment method do you prefer? *

Mark only one oval.

- Intramedullary nail with tumor resection and using Polymethyl methacrylate (PMMA)
- Intramedullary nail without tumor resection
- Plate-screw fixation device
- Modular endoprosthesis with tumor resection
- No indications for surgical treatment
- Other: ____________________

Case 13

A 60-year-old patient
- pathological fracture
- breast cancer with metastases to the shaft of the humerus (confirmed by histopathology)
- severe pain causing disability
- life expectancy over 12 months

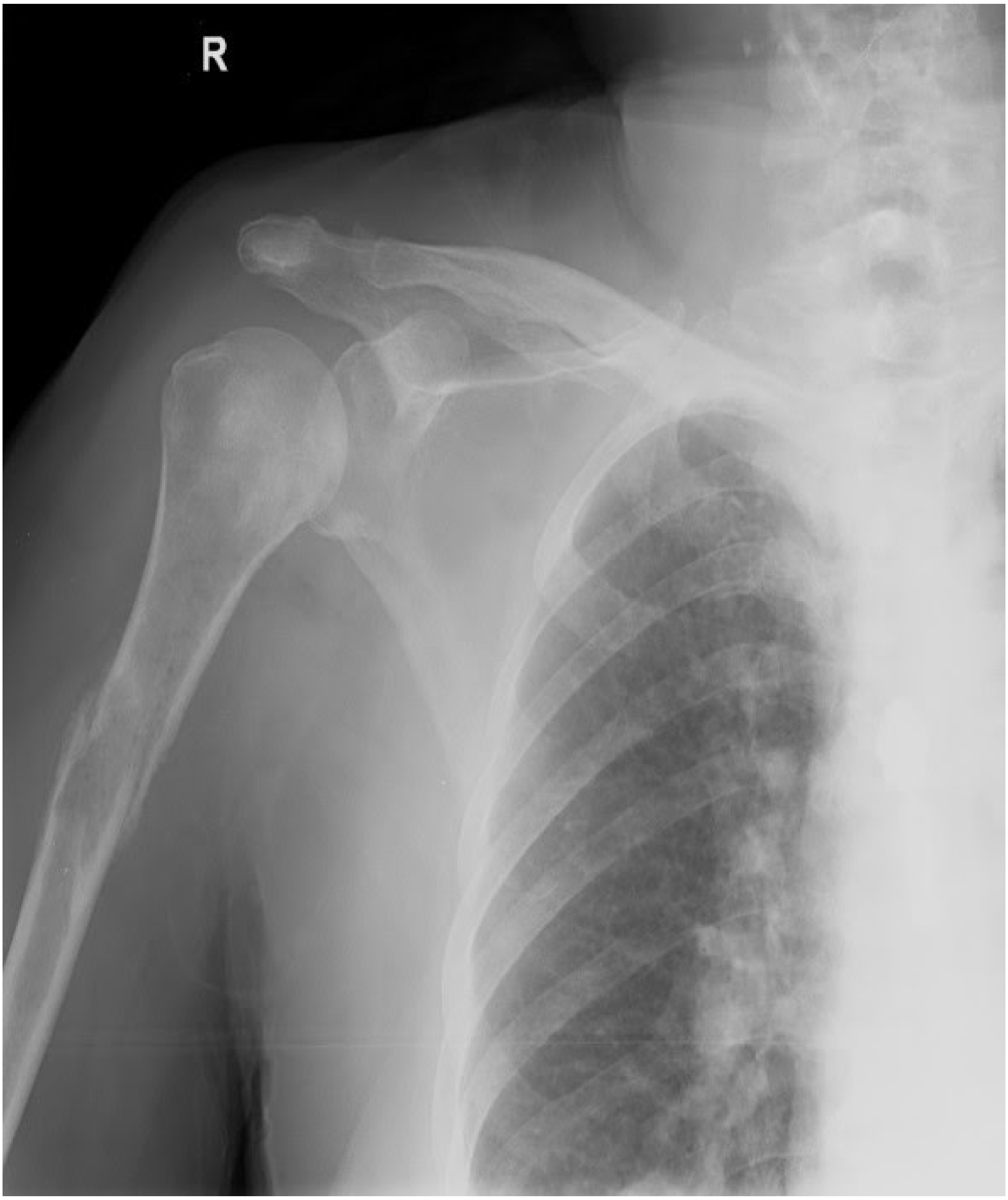
R

40. Which treatment method do you prefer? *

Mark only one oval.

- Intramedullary nail with tumor resection and using Polymethyl methacrylate (PMMA)
- Intramedullary nail without tumor resection
- Plate-screw fixation device
- Modular endoprosthesis with tumor resection
- No indications for surgical treatment
- Other: __________

Case 14

A 60-year-old patient
- pathological fracture
- breast cancer with metastases to the shaft of the humerus (confirmed by histopathology)
- severe pain causing disability
- life expectancy less than 6 months

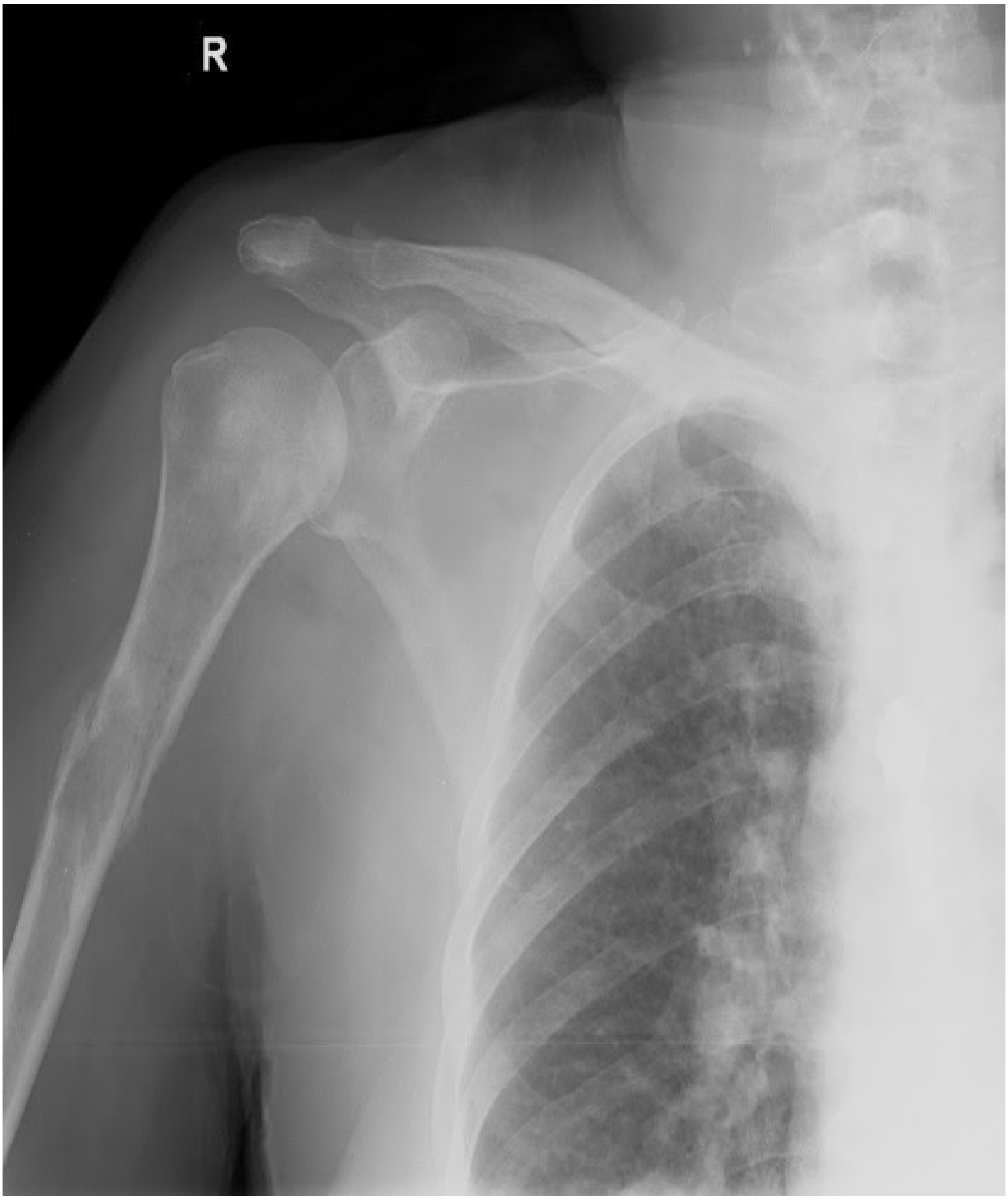
R

41. Which treatment method do you prefer? *

Mark only one oval.

- Intramedullary nail with tumor resection and using Polymethyl methacrylate (PMMA)
- Intramedullary nail without tumor resection
- Plate-screw fixation device
- Modular endoprosthesis with tumor resection
- No indications for surgical treatment
- Other: ____________________

Case 15

A 60-year-old patient
- no evidence of pathological fracture on X-ray, high risk of pathological fracture (impending fracture)
- breast cancer with metastases to the femoral shaft (confirmed by histopathology)
- severe pain causing disability
- life expectancy over 12 months

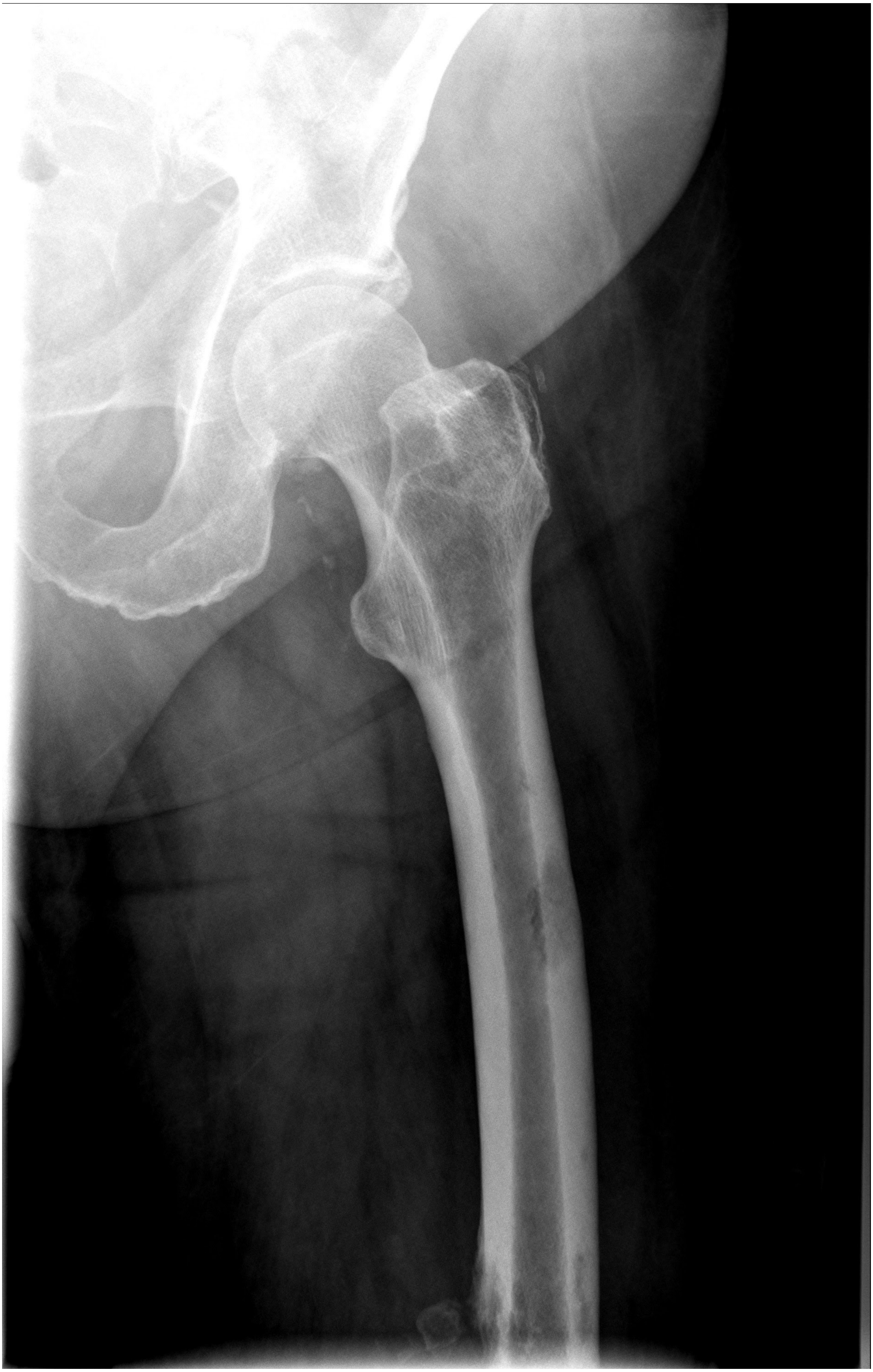

42. Which treatment method do you prefer? *

Mark only one oval.

- Intramedullary nail with tumor resection and using Polymethyl methacrylate (PMMA)
- Intramedullary nail without tumor resection
- Plate-screw fixation device
- Modular endoprosthesis with tumor resection
- No indications for surgical treatment
- Other: ______________________

Case 16

A 60-year-old patient
- no evidence of pathological fracture on X-ray, high risk of pathological fracture (impending fracture)
- breast cancer with metastases to the femoral shaft (confirmed by histopathology)
- severe pain causing disability
- life expectancy less than 6 months

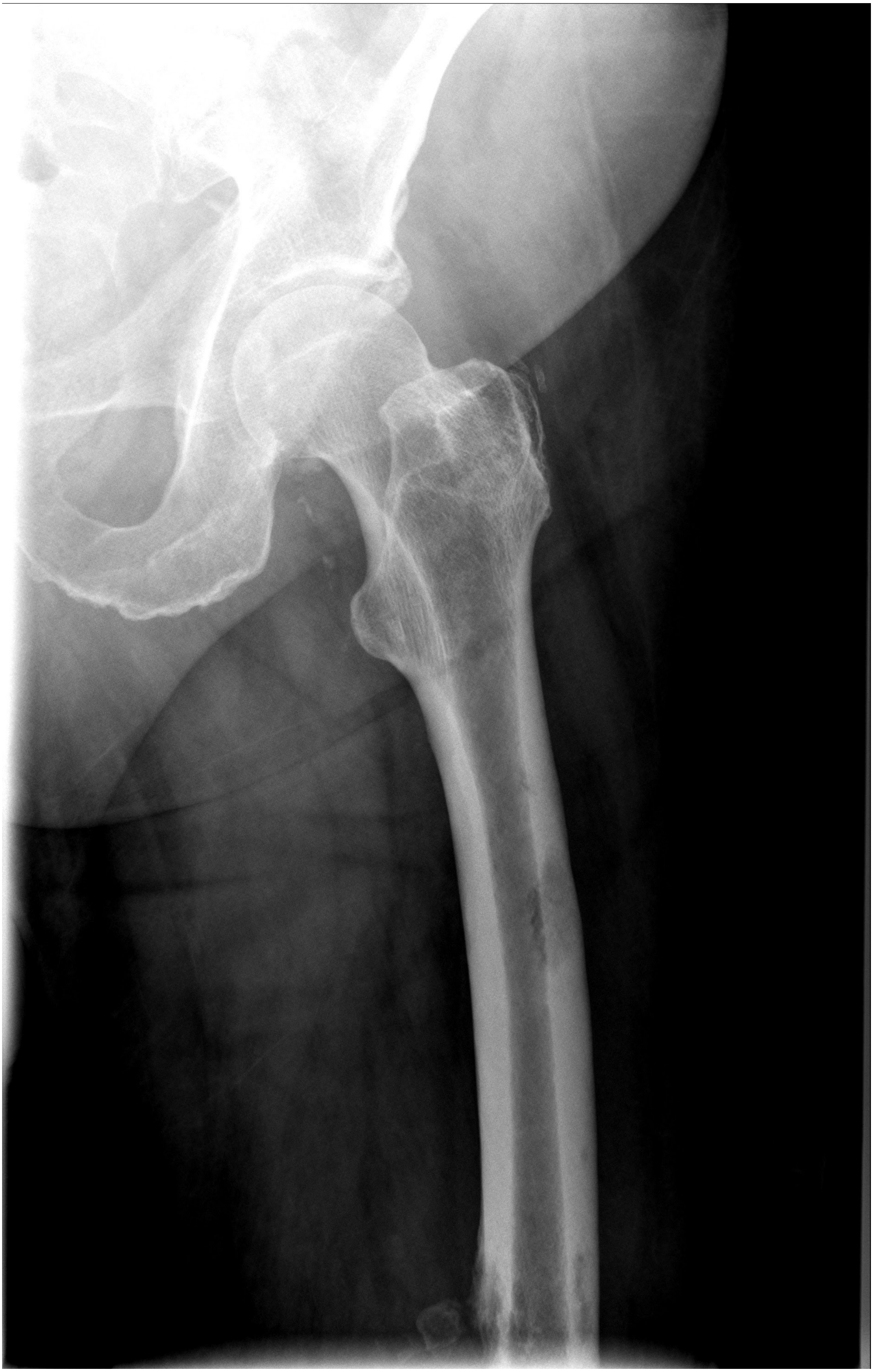

43. Which treatment method do you prefer? *

Mark only one oval.

- Intramedullary nail with tumor resection and using Polymethyl methacrylate (PMMA)
- Intramedullary nail without tumor resection
- Plate-screw fixation device
- Modular endoprosthesis with tumor resection
- No indications for surgical treatment
- Other: ______________________

44. In the case of a patient with a single metastatic lesion of kidney cancer to the femoral shaft, detected by chance (no pain symptoms) with a good prognosis and an timer of> 3 years from the detection of the primary lesion, what would be your preferred method of treatment after excluding primary bone cancer? *

Mark only one oval.

- No indications for surgical treatment
- Intramedullary nail with tumor resection and using Polymethyl methacrylate (PMMA)
- Intramedullary nail without tumor resection
- Plate-screw fixation device
- Modular endoprosthesis with tumor resection
- Other: ______________________

Additional information

45. Thank you very much for completing the survey. If you have any comments, questions or would like to share your experience in dealing with bone metastases in more detail, please complete the field below:

Radiological images - sources

Przypadek 1-2) Case courtesy of Dr Bruno Di Muzio, Radiopaedia.org. From the case rID: 26536
Przypadek 3-4) Case courtesy of Dr Subash Thapa, Radiopaedia.org. From the case rID: 40234
Przypadek 5-6) Case courtesy of Dr Sajoscha Sorrentino, Radiopaedia.org. From the case rID: 16538
Przypadek 7-8) Case courtesy of Dr Ian Bickle, Radiopaedia.org. From the case rID: 75119
Przypadek 9-10) Case courtesy of Assoc Prof Frank Gaillard, Radiopaedia.org. From the case rID: 23325
Przypadek 11-12) Case courtesy of Dr Henry Knipe, Radiopaedia.org. From the case rID: 30462
Przypadek 13-14) Case courtesy of Dr Henry Knipe, Radiopaedia.org. From the case rID: 47200
Przypadek 15-16) Case courtesy of Dr Lawrence Oh, Radiopaedia.org. From the case rID: 28869

This content is neither created nor endorsed by Google.

Google Forms
